# Okamoto model for necrosis and its expansions, CD38-cyclic ADP-ribose signal system for intracellular Ca^2+^ mobilization and Reg (***Re****generating* ***g****ene* protein)-Reg receptor system for cell regeneration

**DOI:** 10.2183/pjab.97.022

**Published:** 2021-10-11

**Authors:** Hiroshi OKAMOTO, Shin TAKASAWA

**Affiliations:** *1 Department of Biochemistry, Tohoku University Graduate School of Medicine, Sendai, Miyagi, Japan.; *2 Department of Biochemistry and Molecular Vascular Biology, Kanazawa University Graduate School of Medical Sciences, Kanazawa, Ishikawa, Japan.; *3 Department of Biochemistry, Nara Medical University, Kashihara, Nara, Japan.

**Keywords:** Langerhans islet β-cells, proinsulin, poly(ADP-ribose) polymerase/synthetase, Okamoto model, cyclic ADP-ribose, *Reg* (***Re**generating **g**ene*)

## Abstract

In pancreatic islet cell culture models and animal models, we studied the molecular mechanisms involved in the development of insulin-dependent diabetes. The diabetogenic agents, alloxan and streptozotocin, caused DNA strand breaks, which in turn activated poly(ADP-ribose) polymerase/synthetase (PARP) to deplete NAD^+^, thereby inhibiting islet β-cell functions such as proinsulin synthesis and ultimately leading to β-cell necrosis. Radical scavengers protected against the formation of DNA strand breaks and inhibition of proinsulin synthesis. Inhibitors of PARP prevented the NAD^+^ depletion, inhibition of proinsulin synthesis and β-cell death. These findings led to the proposed unifying concept for β-cell damage and its prevention (the Okamoto model). The model met one proof with PARP knockout animals and was further extended by the discovery of cyclic ADP-ribose as the second messenger for Ca^2+^ mobilization in glucose-induced insulin secretion and by the identification of *Reg* (***Re****generating* ***g****ene*) for β-cell regeneration. Physiological and pathological events found in pancreatic β-cells have been observed in other cells and tissues.

## 1 Introduction

The islets of Langerhans are located in the pancreas and are the only organs that produce insulin for the target tissues. Loss or dysfunction of the islets results in the development of diseases exemplified by *diabetes mellitus*. Recent advances in molecular biology^1)^ along with improved procedures for isolating the islets of Langerhans from pancreases^2)^ have made it possible to study the insulin synthesis and secretion at the molecular level.

The biosynthesis of insulin in the β-cells of pancreatic islets of Langerhans involves the transcription of proinsulin mRNA from the insulin gene, translation of the proinsulin mRNA by ribosomes associated with rough endoplasmic reticulum, conversion of proinsulin to insulin in the Golgi apparatus, and the formation of β-granules.^3)^ Both proinsulin synthesis and insulin secretion in the islets of Langerhans are known to be markedly induced by glucose.^3)^

Insulin-dependent (Type 1) diabetes is caused by a destructive process affecting the insulin-producing β-cells of the islets of Langerhans. Destruction of β-cells may be induced by inflammatory tissue damage and β-cytotoxins such as alloxan and streptozotocin.

Since the early 1980s, we have proposed a hypothesis concerning β-cell damage and its prevention, according to which poly(ADP-ribose) polymerase/synthetase (PARP) activation induced by DNA strand breaks is critically involved in the consumption of NAD^+^, leading to energy depletion, decreased cell functions including proinsulin synthesis and cell death by necrosis.^2,4–7)^ This hypothesis was reconfirmed by results using PARP knockout (KO) mice^8–11)^ and has been recognized as providing the basis for necrotic cell death of various cells and tissues. Since then, the hypothesis has become known as the Okamoto model for β-cell damage and its prevention.^12–14)^ Based on this model, we have proposed two novel signal systems in β-cells: one is the CD38-cyclic ADP-ribose (cADPR) signal system for insulin secretion, and the other is the ***Re****generating* ***g****ene* protein (Reg)-Reg receptor system for β-cell regeneration. The physiological and pathological significance of the two signal systems in a variety of cells and tissues as well as in pancreatic β-cells has recently been recognized.

Here, we describe the Okamoto model and its expansions, the CD38-cADPR signal system and the Reg-Reg receptor system, focusing on recent advances and how their significance came to light. PARP activation reduces the cellular NAD^+^, leading to decrease the formation of cADPR (which is synthesized from NAD^+^ and is a second messenger for insulin secretion) as well as causing necrotic β-cell death, and PARP is involved in *Reg* gene transcription inducing β-cell regeneration. Therefore, it is reasonable to assume that PARP and its inhibitors have key roles in the prevention of β-cell death, the maintenance of insulin synthesis and secretion, and the induction of β-cell regeneration.

This article shows the achievements gained by a long exploration with the islets of Langerhans, during which we further clarified the physiology of insulin production and the pathogenesis of diabetes and its complications, as described below. In particular, interest in the physiological or pathological event observed in pancreatic β-cells of the islets of Langerhans has been hightened by its possible extension to other cells and tissues, as shown in Figs. 5, 6, 11, 14, 17, 18, 19, 20, 21, and 22 in this article.

## 2 Isolation of the islets of Langerhans from pancreas

**Figure 1.**
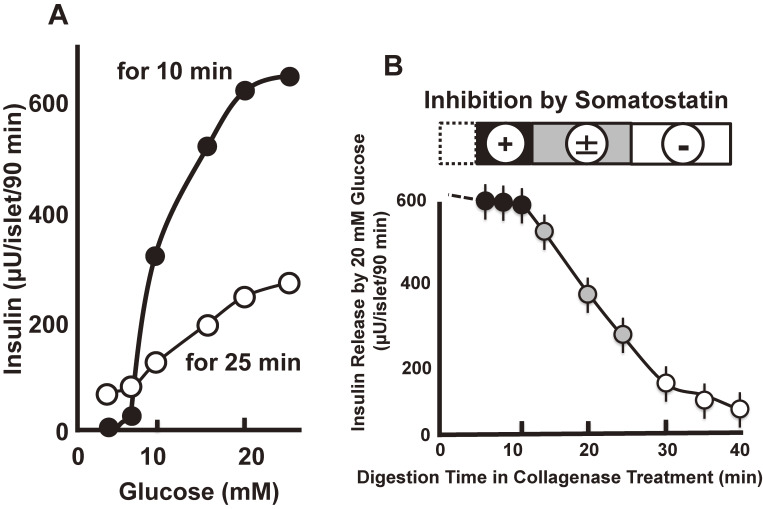
Insulin release from isolated pancreatic islets (adapted from Ref. 2). **A.** Rat pancreatic islets were isolated by the method of Okamoto *et al.*^2,16)^ The collagenase treatment was carried out for 10 min (closed circle) or 25 min (open circle). The insulin released from isolated islets was examined as described previously.^16,287)^ **B.** Effect of varying periods of collagenase treatment on islet insulin release and on its susceptibility to somatostatin. Results represent the mean ± S.E. in µU of insulin released in 4 incubation flasks. The flask contained 6 isolated islets. +: The insulin release was inhibited by somatostatin. −: The insulin release was not inhibited by somatostatin. ±: Inhibition by somatostatin of insulin release was not prominent.

The procedure for isolating the islets of Langerhans from rodent pancreas was established by Lacy and Kostianovsky.^15)^ This group introduced collagenase digestion to dissect endocrine tissues in the pancreas from the surrounding exocrine tissues. However, the possibility that some functions of the islets are impaired during the isolation may not be totally ignored. Nevertheless, little attention has been fixed on this possibility. We at first examined the possible impairment of the islet functions by determining the insulin secreting ability, since if any damage occurred to islets during the isolation process, it would be reflected first by the insulin secreting ability rather than by the insulin synthesizing function. Okamoto *et al.*^2,16)^ improved the method of isolating rat islets by adding bovine serum albumin to the medium for collagenase-treatment of rat pancreas and employing a Buchler’s vigorously rotatory incubator-shaker. As shown in Fig. 1A, compared to the islets obtained after 25 min treatment, those obtained after 10 min treatment exhibited a steeper response of insulin secretion to glucose stimulation. The islets obtained after a relatively brief treatment with collagenase also displayed a distinct susceptibility to somatostatin, which was no longer observed in islets obtained after relatively long treatment. Somatostatin (10 ng/mL) inhibited insulin secretion from the islets obtained after 10 min collagenase treatment by approximately 50%, whereas the insulin secretion from islets obtained after 25 min treatment was not affected at all by the somatostatin (Fig. 1B). The modified method consistently yielded hundreds of intact islets per one pancreas. The amount of insulin secreted reached approximately 600 µU/islet/90 min as the treatment period decreased. We were thus led to conclude that the value obtained by extrapolation of the treatment period to 0 min gives the amount of insulin secreted from an ‘intact’ islet. Therefore, that almost ‘intact’ pancreatic islets were obtained was confirmed by examining the amount of insulin secreted from an isolated islet, the response curve of insulin secreted corresponding to change in glucose concentration, and the sensitivity to somatostatin.

It should be noted here that somatostatin inhibited the glucose-induced insulin secretion from the islets by at most 50% and the inhibitory effect of somatostatin was not significant when the Ca^2+^ concentration in the incubation medium was raised from 5 to 10 mEq/L.^2,16)^ These results also suggested the importance of Ca^2+^ mobilization from the intracellular Ca^2+^ pool in the insulin secretion as well as from the extracellular Ca^2+^ influx, as described in Sections 5 and 7.

## 3 Translational control of proinsulin synthesis by glucose in the islets of Langerhans

**Figure 2.**
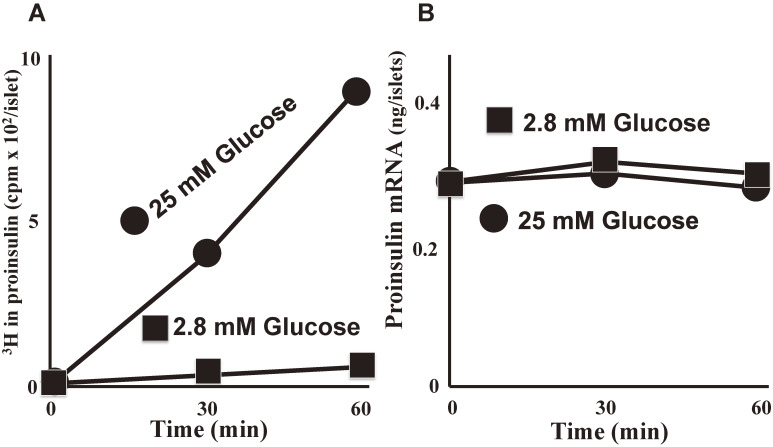
Proinsulin mRNA levels and the time course of induction of proinsulin synthesis by glucose (adapted from Ref. 17). Rat pancreatic islets were incubated at 37 ℃ in the presence of 2.8 mM (filled squares) or 25 mM (filled circles) glucose and [^3^H]leucine. **A.** [^3^H]Leucine-labeled products in the islets were analyzed by SDS-polyacrylamide gel electrophoresis and the amount of *de novo* synthesized proinsulin was determined by summing ^3^H-radioactivity of the gel slices corresponding to the proinsulin peak. **B.** Aliquots of the nucleic acids from the incubated islets were used for the quantification of proinsulin mRNA by hybridization with proinsulin cDNA.

Proinsulin synthesis in the islets of Langerhans of rats as well as in many other animals is known to be induced by glucose,^3)^ in marked contrast to non-insulin proteins, the synthesis of which is only slightly affected by glucose.^2)^ Thus, there must be a highly selective mechanism in the islets for proinsulin synthesis, either at the level of insulin gene transcription or at the level of proinsulin mRNA translation. If proinsulin induction by glucose is regulated at the transcriptional level, the amount of proinsulin mRNA may be increased by the administration of glucose. Alternatively, if proinsulin induction is regulated at the translational level, the rate of proinsulin synthesis may not be correlated with the amount of proinsulin mRNA. Itoh and Okamoto^17)^ assayed the *de novo* synthesis of proinsulin with concomitant quantification of proinsulin mRNA in the islets of Langerhans, using nucleic acid hybridization with cDNA synthesized from purified rat proinsulin mRNA.^2,17,18)^ The approach revealed that, while the islet synthesis of proinsulin is significantly stimulated by exposure to high glucose, the amount of proinsulin mRNA in the glucose-stimulated or unstimulated islets remained unchanged during the period of proinsulin induction up to 60 min (Fig. 2). In a wheat-germ cell-free system, the amount of proinsulin mRNA in islets as determined by the *in vitro* translation assay remained unchanged during the period of proinsulin induction by glucose.^19,20)^

We examined the transcriptional activity of the insulin gene in rat islets using recombinant DNA containing a rat proinsulin cDNA sequence as a probe. Although proinsulin synthesis was stimulated 8.4-fold by glucose, proinsulin mRNA synthesis was stimulated only 1.8-fold.^21,22)^ Cycloheximide, an inhibitor of eukaryotic translation elongation, completely inhibited the glucose-induced rise in proinsulin synthesis. α-Amanitin, an inhibitor of RNA polymerase II (weakly RNA polymerase III), had essentially no effect on the proinsulin mRNA level in pancreatic islets of rats.^21,22)^ These results clearly indicate that proinsulin synthesis is regulated at the translational level (translational control).^2,17)^

As insulin regulates the glucose level in the blood, the rates of both insulin synthesis and insulin secretion need to change rapidly according to the change in the blood glucose concentrations. This regulation of proinsulin synthesis should be the means by which the rapid regulation of insulin synthesis by glucose is ensured.^23)^ In fact, Itoh^23)^ estimated the number of proinsulin mRNA molecules per rat pancreatic islet β-cell to be approximately 2.7 × 10^5^. The number of proinsulin mRNA molecules per rat pancreatic β-cell was comparable to that of globin mRNA per rabbit reticulocyte^24)^ (1.4 × 10^5^ per cell) and ovalbumin mRNA per chicken oviduct cell^25)^ (1.5 × 10^5^ per cell), which had been thought to represent the most abundant mRNA species in eukaryotic cells. The control of proinsulin synthesis was assumed to be achieved by the selective enhancement of the translation of proinsulin mRNA at the elongation step.^17,22)^ The 5′-untranslated region of proinsulin mRNA was reported to be necessary for the glucose-induced translational control in mouse and human β-cells.^26,27)^

There are some well-documented examples of the translational control of specific gene products. In such examples as ornithine aminotransferase,^28)^ ornithine decarboxylase^29)^ and ferritin,^30)^ translational control is regulated at the translation initiation step. The translational control of proinsulin synthesis, which seems to be regulated mainly at the elongation step, is a unique regulation of gene expression in eukaryotic cells.

## 4 The Okamoto model for β-cell damage and its prevention

Diabetes can be usefully studied at an experimental level by methods that make use of either surgical techniques or chemical agents.

Many of the acute metabolic derangements of severe human insulinopenic diabetes can be reproduced by removing the insulin-producing pancreatic β-cells of the islets of Langerhans. This artificial form of diabetes was first produced by von Mering and Minkowski^31)^ when they removed the pancreases of dogs. The same model of diabetes was adopted by Banting and Best^32)^ for their historic observation on the hypoglycemic properties of a crude pancreatic extract of insulin.

For chemical agents, alloxan^33)^ (2,4,5,6-tetraoxohexahydropyrimidine) and streptozotocin^34)^ (2-deoxy-2-(3-methyl-3-nitrosoureido)-D-glucopyranose) are particularly instructive; they exert selective cytotoxic effects on pancreatic β-cells in animals and are extremely potent diabetogenic substances. Since the original discoveries were made by Dunn *et al.*^33)^ and Rakieten *et al.*,^34)^ alloxan and streptozotocin have been widely used to induce diabetes in experimental animals because of their advantages in terms of specificity, convenience and reproducibility. How the β-cytotoxins cause functional deteriorations and degenerative changes in the β-cells, however, had been the subject of much debate, until we proposed a unifying concept for the diabetogenicity of alloxan and streptozotocin.^2,4–7,35–37)^

### 4.1 Alloxan and streptozotocin induce DNA strand breaks and poly(ADP-ribose) polymerase/synthetase (PARP) that depletes NAD^+^ in pancreatic islets.

**Figure 3.**
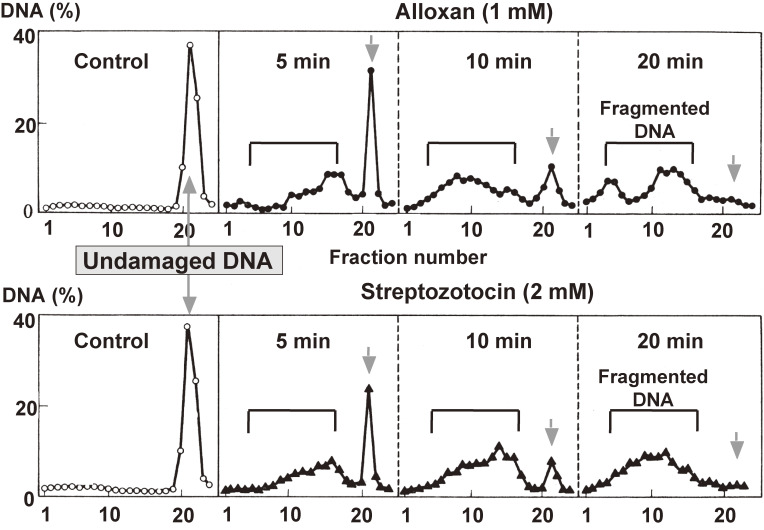
Islet DNA strand breaks by alloxan (circles: upper panel) and streptozotocin (triangles: lower panel) (adapted from Ref. 4). Islets isolated from rat pancreas were incubated with alloxan or streptozotocin for 5–20 min in Krebs-Ringer’s bicarbonate medium. After incubation, DNA from islets that were laid for 10 min without the diabetogenic agents was recovered as a single peak near the bottom of the sucrose gradient, the position at which was undamaged DNA sediments. However, after only 5 min incubation with 1 mM of alloxan or 2 mM of streptozotocin, a considerable amount of DNA was deposited as a broad peak in the middle of the gradient with a concomitant decrease in undamaged DNA; after 10–20 min incubation, the DNA was almost completely fragmented. The effect of the two agents on islet DNA fragmentation was dose-dependent. These results indicate that alloxan and streptozotocin produce strand breaks in islet DNA.

In 1981, Yamamoto *et al.*^4)^ demonstrated that streptozotocin and alloxan cause DNA strand breaks in isolated rat islets. As shown in Fig. 3, DNA from intact islets incubated in the absence of the diabetogenic agents was recovered as a single peak near the bottom of the alkaline sucrose gradient, the position at which undamaged DNA is deposited as a sediment. However, after only 5 min incubation with streptozotocin or alloxan, a considerable amount of DNA is deposited as a broad peak in the middle of the gradient with a concomitant decrease in undamaged DNA; after 10–20 min incubation, the islet DNA was almost completely fragmented. The results indicated that streptozotocin and alloxan produce strand breaks in islet DNA.

**Figure 4.**
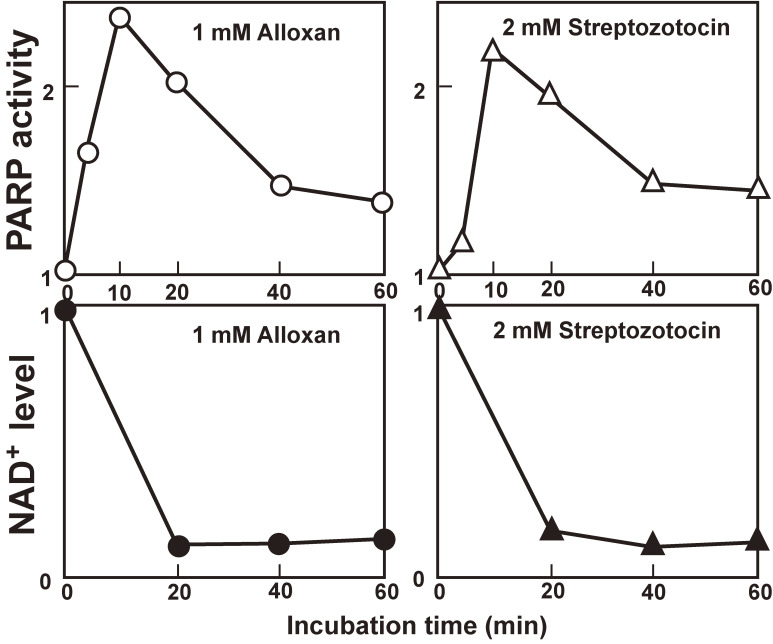
Effect of alloxan and streptozotocin on pancreatic islets (adapted from Ref. 4). Nuclear fraction from islets was incubated in conditions causing breaks in islet DNA, and PARP activity was assayed (*Upper*). Both streptozotocin (2 mM) and alloxan (1 mM) induced a 2- to 3-fold increase in PARP activity, with a peak at 10 min. Cellular NAD^+^ content was reduced by either 2 mM streptozotocin or 1 mM alloxan within 20 min of incubation and remained almost unaltered for 60 min (*Lower*). There was a striking temporal correlation between the decrease in the level of islet NAD^+^ and the increase in islet PARP activity.

Next, Yamamoto *et al.*^4)^ prepared a nuclear fraction from islets incubated in conditions that cause the DNA breaks, and assayed the activity of PARP, a nuclear enzyme that catalyzes the polymerization of the ADP-ribosyl moiety of NAD^+^ to form poly(ADP-ribose); the enzyme was described by three independent groups in France and Japan, namely, Chambon *et al.*,^38)^ Sugimura *et al.*^39)^ and Nishizuka *et al.*,^40)^ and was implicated in DNA repair.^41)^ Both streptozotocin and alloxan were found to induce islet PARP activity with a peak at 10 min (Fig. 4 upper panel). The increase in nuclear PARP activity was associated with a concomitant decrease in cellular NAD^+^, the substrate of PARP; the NAD^+^ content of the islets showed a sharp drop within 20 min of incubation with either streptozotocin or alloxan (Fig. 4 lower panel).

The diabetogenic doses of alloxan and streptozotocin did induce *in vivo* DNA strand breaks and NAD^+^ depletion in the islets of Langerhans.^5)^ From these results, it is reasonable to assume that the β-cytotoxins cause DNA breaks to induce PARP, thereby depleting islet NAD^+^.

Human genome sequence projects revealed the occurrence of similar DNA sequences to PARP. At least seventeen PARP-related genes (PARP-1, -2, -3, -4, -5a, -5b, -5c, -6, -7, -8, -9, -10, -11, -12, -14, -15, and -16) were revealed to constitute a multigene family, the PARP gene family.^42–44)^ PARP-1 activation by extensive DNA damage is the major pathway in necrotic cell death, and therefore “PARP” will be used from here on to indicate “PARP-1”.

### 4.2 Protection by radical scavengers and PARP inhibitors against alloxan- and streptozotocin-induced islet DNA strand breaks, the NAD^+^ depletion and the inhibition of proinsulin synthesis.

Yamamoto *et al.*^4)^ incubated isolated rat pancreatic islets in the presence of alloxan or streptozotocin with or without the addition of PARP inhibitors such as nicotinamide and picolinamide. The inhibitors almost completely abolished the alloxan- and streptozotocin-induced decreases in the islet proinsulin synthesis as well as in the islet NAD^+^ level.^4)^ The various PARP inhibitors (benzamide, 3-aminobenzamide, 3-nitrobenzamide, 3-methoxybenzamide, picolinamide, nicotinamide, theophylline, and IBMX) were found to reverse the alloxan- or streptozotocin-induced inhibition of proinsulin synthesis in a dose-dependent manner, and the stronger inhibitors exerted protective effects at lower concentrations.^1,6,36)^ *In vivo* administrations of nicotinamide or 3-aminobenzamide to rats effectively protected against the alloxan- or streptozotocin-induced decrease in the islet ability to synthesize proinsulin.^7)^

It has been suggested that alloxan may work through the formation of the hydroxyl radical (OH^•^),^45)^ which is produced by the interaction between superoxide (O_2_^−•^) and peroxide (H_2_O_2_)^46)^:

$$\text{O}{}_{2}{}^{-∙}+\text{H}{}_{2}\text{O}{}_{2}\to \text{OH}{}^{∙}+\text{OH}{}^{-}+\text{O}{}_{2}$$

Superoxide dismutase and catalase catalyze the removal of O_2_^−•^ and H_2_O_2_, respectively,^47)^ and hence may inhibit the formation of OH^•^. Uchigata *et al.*^6)^ showed that combined administration of superoxide dismutase and catalase more effectively protects against alloxan-induced islet DNA strand breaks, as well as against proinsulin synthesis inhibition, than administration of either of these scavenging enzymes alone. The results indicate that it is OH^•^ that is ultimately generated from alloxan to attack DNA. On the other hand, streptozotocin-induced islet DNA strand breaks were not affected by the radical scavengers.^7)^ The breakage of DNA by streptozotocin is probably associated with its alkylating activity, as suggested with nitrosoureas.^48)^

### 4.3 The proposal of the Okamoto model for β-cell damage and its prevention.

**Figure 5.**
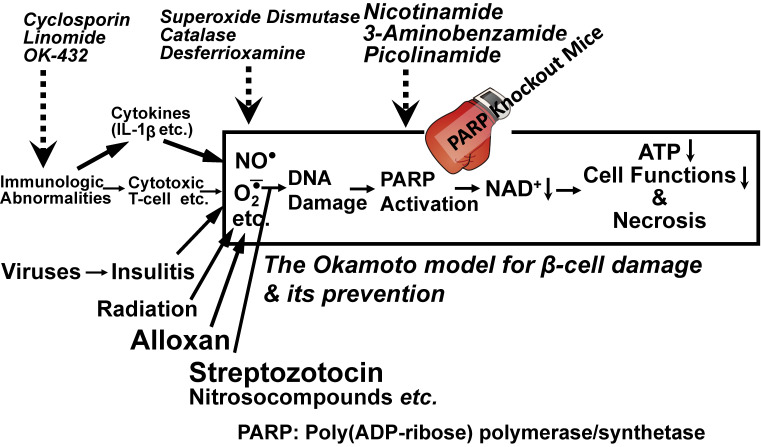
(Color online) The Okamoto model for β-cell damage and its prevention (adapted from Refs. 2, 36, 37, 96 and 101). Although Type 1 diabetes can be caused by many different agents such as immunological abnormalities, inflammatory tissue damage, alloxan and streptozotocin, the final pathway leading to β-cell damage is the same. This pathway involves the generation of free radicals, DNA damage, nuclear PARP activation and NAD^+^ depletion. Therefore, the β-cell damage is theoretically preventable through inhibition of the serial reactions, as indicated by dashed arrows. One method is by inhibiting abnormal immune reactions with immunomodulators such as cyclosporin, linomide, and OK-432.^58,59)^ Others are scavenging the radicals, which break DNA, by superoxide dismutase, catalase, and desferrioxamine^12)^ and inhibiting the PARP by specific inhibitors such as nicotinamide, 3-aminobenzamide, and picolinamide to prevent the decrease in the NAD^+^ level. The unifying concept for β-cell damage and its prevention explains both how autoimmunity for β-cell necrosis is initiated and how necrotic cell death is involved in various diseases in many tissues other than β-cells.^63–74,96)^

From the experimental results described above, Okamoto *et al.*^2,6,14,36,37)^ proposed a basic model for the action of alloxan and streptozotocin in the induction of experimental diabetes. As shown in Fig. 5, the first step is the generation of free radicals by alloxan and streptozotocin, which attack DNA to produce strand breaks. PARP then acts to repair the DNA breaks,^2,36,37)^ consuming β-cell NAD^+^. Since NAD^+^ is the most abundant of cellular coenzymes and participates in many biological reactions in the cell, a severe reduction in intracellular NAD^+^ to non-physiological levels can adversely affect β-cell functions, including ATP production, proinsulin synthesis and insulin secretion, and thus the β-cell ultimately dies. Therefore, in diabetes induction, the β-cells seem to be making “a suicide response” in their attempt to repair the damaged DNA.^2,36,37)^

Here the problem arises as to why only pancreatic β-cells are specifically damaged by alloxan and streptozotocin. It has been conjectured that alloxan and streptozotocin have an affinity to the cell membranes of β-cells because of their chemical structures.^49)^ ^14^C-Labeled alloxan or streptozotocin injected into mice was recovered in pancreatic islets.^50,51)^ The NAD^+^ content per DNA of normal pancreatic islets was approximately one half of that of the liver^5)^ and, therefore, pancreatic β-cells may be more susceptible to a reduction in NAD^+^ levels. Malaisse *et al.*^52)^ reported that the ability to provide protection against potent reactive radicals may be weak in islet cells given the low glutathione peroxidase activity in islets.

This model for the mechanism of action of alloxan and streptozotocin has received much attention because of its possible relevance to the effects of viruses and inflammation, especially those related to inflammatory damage of β-cells,^2,14,36,37)^ because biological events like inflammation and virus infection, physical insults like radiation, and chemical insults may independently or interactively produce β-cell DNA strand breaks. Repeated administration of subdiabetogenic doses of streptozotocin to rats induced insulitis and a diabetic state similar to Type 1 diabetes.^53)^ Tsubouchi *et al.*^54)^ have reported that a large dose of X-ray irradiation provoked the necrosis of pancreatic islets in hamsters, and that the hamsters exhibited a diabetic state. Cumulative β-cell damage induced by subdiabetogenic doses of streptozotocin and by encephalomyocarditis or Coxsackie virus infection was shown to result in the development of diabetes.^55)^

Therefore, although insulin-dependent Type 1 diabetes can be caused by a variety of internal and external environmental factors, including immunologic abnormalities, inflammatory tissue damage, viruses, irradiation, and chemical insults, the final pathway may be one and the same (Fig. 5), involving DNA damage, PARP activation, and NAD^+^ depletion. Accordingly, it may be possible to prevent insulin-dependent Type 1 diabetes by blocking immune reactions, scavenging free radicals, and inhibiting PARP. Actually, diabetes in non-obese diabetic (NOD) mice,^56)^ a murine model that spontaneously develops clinical and pathological manifestations similar to those seen in human Type 1 diabetes, is reported to be preventable by treatment with immunosuppressive agents,^57)^ an immunomodulator OK-432,^58,59)^ radical scavengers,^12)^ and PARP inhibitors.^12,60)^ Concerning nitric oxide (NO) (see Fig. 5), Takamura *et al.*^61)^ found that in transgenic mice expressing type 2 nitric oxide synthase (NOS2) constitutively in pancreatic β-cells, the β-cell mass was markedly reduced resulting in the development of severe diabetes. NOS2 is usually induced by proinflammatory/inflammatory cytokines such as interleukin-1β, interferon-γ, and tumor necrosis factor-α.

### 4.4 Proof and extension of the Okamoto model.

In 1999, three independent groups in Germany, Japan and the U.S. provided irrefutable support using PARP deficient mice for the model shown in Fig. 5. The mice were extremely resistant to streptozotocin and the β-cell death was prevented.^8–11)^ PARP deficient mice were resistant against not only single-high-dose streptozotocin-induced diabetes, as described above, but also multiple-low-dose streptozotocin-induced diabetes.^62)^

PARP is one of the best known proteins with DNA-damage scanning activity, and poly(ADP-ribosyl)ation by PARP has been proposed to function in DNA repair by modifying architectural proteins proximal to DNA breaks, thus facilitating the opening of the condensed chromatin structure required for the recruitment of the repairing complex.^41)^ Paradoxically, in spite of this beneficial effect, PARP can induce necrotic cell death (“necrosis”) through NAD^+^ depletion as described above. In spite of the apparent Mr. Hyde-like side of this enzyme, it may represent an evolutionary strategy adopted by multicellular organisms to prevent the survival of cells that would otherwise transmit potentially dangerous genetic material. Many other tissues and cells such as those involved in cerebral ischemia,^63)^ myocardial ischemia,^64)^ abdominal aortic aneurysm,^65)^ laryngeal injury,^66)^ intestinal mucosal injury,^67)^ organ injury by hemorrhagic shock,^68)^ ischemic renal injury,^69)^ acute pancreatitis,^70)^ acetoaminophen-induced liver cell death,^71)^ benzo[a]pyrene-induced liver cell death,^72)^ diabetic kidney disease,^73)^ and diabetic myocardial and endothelial injury^74)^ have been reported to die by the same mechanism as that in pancreatic β-cell death (see Fig. 5). Accordingly, the inhibition of PARP activity may be a possible therapeutic approach in a wide number of disorders other than diabetes.

## 5 The CD38-cyclic ADP-ribose (cADPR) signal system for glucose-induced insulin secretion in pancreatic β-cells of the islets of Langerhans

Glucose increases the intracellular Ca^2+^ concentration in pancreatic β-cells to cause the secretion of insulin. Ashcroft *et al.*^75)^ proposed in 1984 that this increase in the Ca^2+^ concentration is provided extracellularly. That is, in the process of glucose metabolism, the millimolar concentrations of ATP produced inhibit the potassium (K_ATP_) channel, causing membrane depolarization and the opening of the voltage-dependent Ca^2+^ channels. In 1995, K_ATP_ channels were reconstituted *in vitro*, and the molecular structure of the channels was determined.^76)^ K_ATP_ channels are hetero-octameric proteins composed of pore-forming Kir6.x subunits and regulatory sulfonylurea receptor (SUR) subunits.^76,77)^

**Figure 6.**
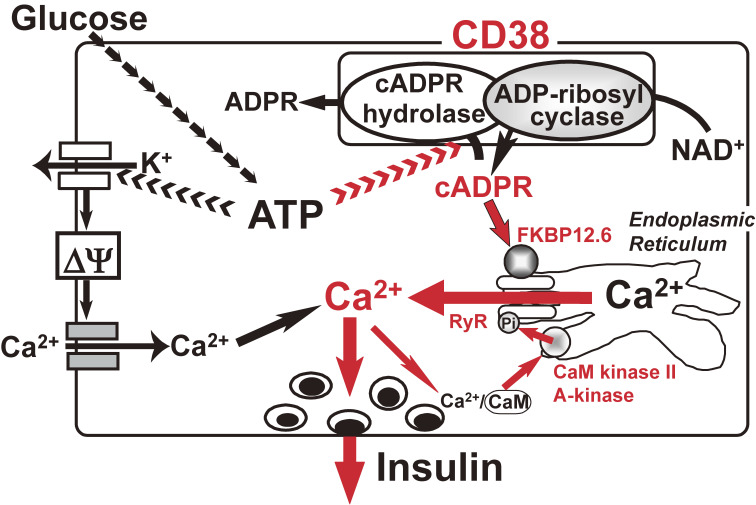
The CD38-cADPR signal system for insulin secretion by glucose stimulation in β-cells (adapted from Ref. 94). Shown in *red* is the regulatory pathway for glucose-induced insulin secretion via the CD38-cADPR signal system. ATP competes with cADPR for the binding site (Lys-129, see also Section 5.2), inhibiting the cADPR hydrolysis which causes the accumulation of cADPR. cADPR binds to FKBP12.6 to release Ca^2+^, dissociating FKBP12.6 from RyR. CaM kinase II phosphorylates RyR to sensitize and activate the Ca^2+^ channel (Pi, phosphorylation). Ca^2+^, released from intracellular stores and/or supplied from extracellular sources, further activates CaM kinase II and amplifies the process. In this way, Ca^2+^-induced Ca^2+^ release may be explained.^288)^ The conventional insulin secretion mechanism by Ca^2+^ influx from extracellular sources is shown in *black*. CaM, calmodulin; RyR, ryanodine receptor; ADPR, ADP-ribose; A-kinase, cyclic AMP-dependent protein kinase.

In a series of studies beginning in 1992^78–103)^ (see also Ref. 104), we proposed another model of insulin secretion by glucose, as shown in Fig. 6 in red; ATP, produced in the process of glucose metabolism, inhibits the cADPR hydrolase of CD38, causing the accumulation of cADPR, which acts as a second messenger for Ca^2+^ mobilization from an intracellular Ca^2+^ pool, the endoplasmic reticulum, for insulin secretion.

### 5.1 Cyclic ADP-ribose, a second messenger for intracellular Ca^2+^ mobilization in glucose-induced insulin secretion.

**Figure 7.**
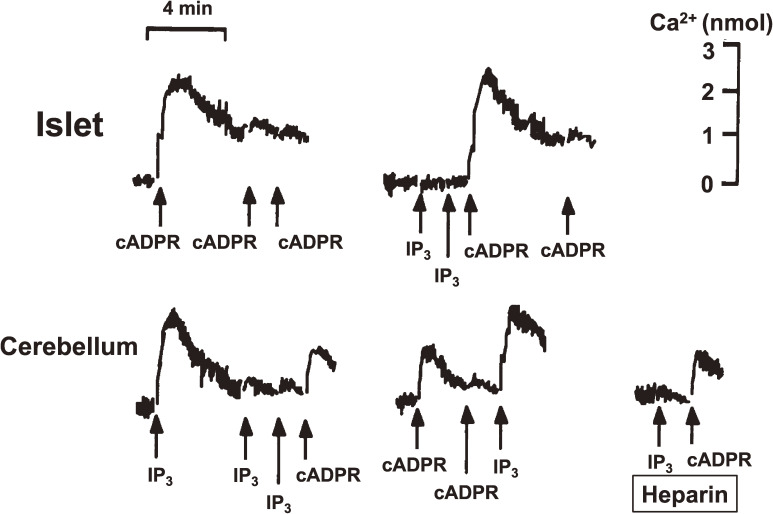
Ca^2+^ release induced by cADPR and IP_3_ from islet and cerebellum microsomes measured fluorometrically with fluo 3 (adapted from Ref. 79). cADPR (1.0 µM), IP_3_ (1.0 µM) and heparin (100 µg/mL) were added as indicated. Fluo 3 fluorescence was measured at 490 nm excitation and 535 nm emission. The absolute amount of Ca^2+^ released is indicated on the ordinate. Breaks in the record occurred during additions to the cuvette.

**Figure 8.**
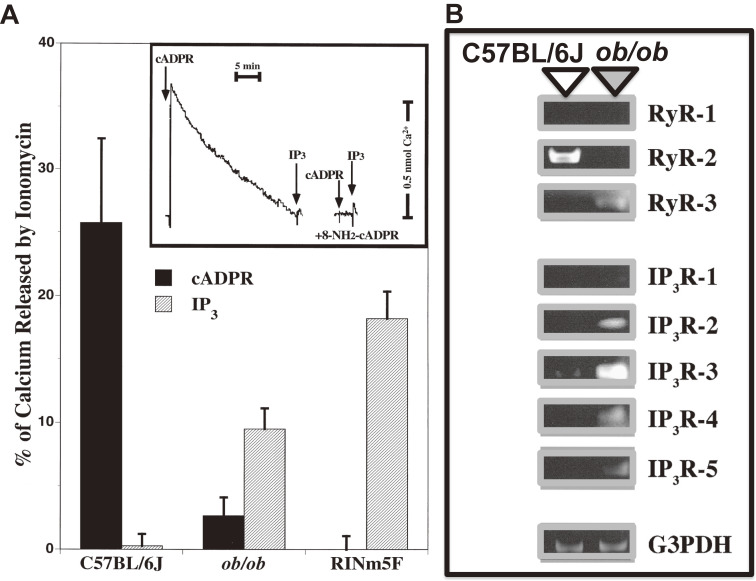
Calcium mobilization by cADPR and IP_3_ from microsomes of C57BL/6J, *ob*/*ob* islets, and RINm5F β-cells (adapted from Ref. 89). **A.** Ca^2+^ release from microsomes (10 µg of protein) was induced by the addition of 100 nM cADPR or 1 µM IP_3_ in the presence of 7 µg/mL calmodulin.^83,103)^ Values are mean ± S.E. of triplicate experiments. The inset shows a typical result of Ca^2+^ release from C57BL/6J mouse islet microsomes. The Ca^2+^ release by cADPR was not affected by the pre-addition of IP_3_, as seen in rat islet microsomes.^89)^ **B.** RT-PCR analyses of C57BL/6J and *ob*/*ob* islets. Expression of RyR and IP_3_R mRNAs.

Cyclic ADP-ribose (cADPR) is a cyclic compound synthesized from NAD^+^. This compound was first found in 1987 by Lee and associates^105)^ when studying Ca^2+^ release in sea urchin eggs. However, the physiological significance of cADPR in mammalian systems was not then understood. According to the Okamoto model, the decrease in the NAD^+^ level should cause a decrease in cADPR in β-cells. Thus, in 1993, we^79,80)^ proposed that insulin secretion by glucose occurred via cADPR-mediated Ca^2+^ mobilization from an intracellular Ca^2+^ pool, the endoplasmic reticulum (Fig. 6). Using rat islet microsomes as a cell-free system to study Ca^2+^ release, cADPR was found to induce Ca^2+^ release from islet microsomes as indicated by the observed prompt increase in fluo 3 (1-[2-Amino-5-(2,7-dichloro-6-hydroxy-3-oxo-9-xanthenyl)phenoxy]-2-(2-amino-5-methylphenoxy)ethane-*N*,*N*,*N*′,*N*′-tetraacetic acid) fluorescence (Fig. 7). Repeated additions of cADPR resulted in attenuation of the Ca^2+^-release response to cADPR. IP_3_ was not found to release Ca^2+^ from islet microsomes, and at this point islet microsomes were still responsive to cADPR. In cerebellum microsomes, IP_3_ induced Ca^2+^ release from the microsomes, and repeated additions of IP_3_ resulted in attenuation of the Ca^2+^-release response. cADPR also induced Ca^2+^ release from cerebellum microsomes, and repeated additions of cADPR resulted in attenuation of the Ca^2+^-release response to cADPR. At this point, cerebellum microsomes were still responsive to the addition of IP_3_. Moreover, heparin, an inhibitor of IP_3_ binding to its receptor, blocked IP_3_-induced Ca^2+^ release. The cADPR-induced Ca^2+^ release from islet microsomes showed a steep dose-response relationship with a clear effect at concentrations as low as 0.1 µM and a near-maximum release at 0.5 µM.^79)^ However, IP_3_ was not found to cause Ca^2+^ release from islet microsomes at these concentrations. These results indicated that islet microsomes did respond to cADPR but not to IP_3_. In contrast with islet microsomes, cerebellum microsomes responded to both cADPR and IP_3_, but cADPR induced Ca^2+^ release from cerebellum microsomes via a different mechanism than that utilized by IP_3_. These results suggested that responses to cADPR and IP_3_ varied according to the tissue or cell type (see also Section 5.5 and Fig. 8).

In Section 4, we explain that diabetogenic agents such as streptozotocin deplete intracellular NAD^+^ in islets. PARP inhibitors such as nicotinamide and 3-aminobenzamide prevent the NAD^+^ depletion through PARP.^2,4,6,36,37)^ We therefore incubated islets with glucose and streptozotocin, prepared the islet extract and measured its Ca^2+^ mobilizing activity.^79)^ A prominent Ca^2+^ release was found with the extract from islets treated with 20 mM glucose, but not with extracts from islets treated with 2.8 mM glucose. Streptozotocin caused a great reduction in the Ca^2+^ mobilizing activity of the extract from islet treated with 20 mM glucose, and PARP inhibitors such as nicotinamide and 3-aminobenzamide reversed the reduction. The results also suggested that the active component for Ca^2+^ release in the glucose-stimulated islet extract was cADPR. In fact, we measured the cADPR content in normal rat and mouse islets by radioimmunoassay with an anti-cADPR antibody and found an increase in the cADPR content by glucose in the islets.^89)^ The increase in the cADPR concentration in response to high glucose was confirmed using BALB/c mouse islets.^106)^

Moreover, cADPR stimulated insulin secretion from digitonin-permeabilized islets.^79)^ Near maximal secretion of insulin by cADPR was observed at 0.5 µM and half-maximal secretion at 0.1 µM. The dose-response curve was well fitted to that of Ca^2+^ release from islet microsomes. In contrast with cADPR, IP_3_ did not induce insulin secretion.

### 5.2 CD38 as a major enzyme for the cADPR synthesis.

**Figure 9.**
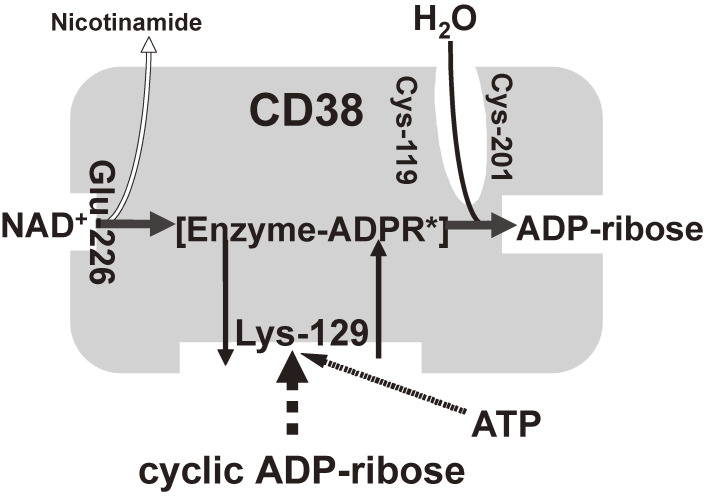
Important amino acids in the cADPR metabolism in CD38 (adapted from Ref. 86). CD38 catalyzes the formation of cADPR from NAD^+^ and hydrolysis of cADPR to ADPR.^80)^ Lys-129 of CD38 is the cADPR binding site and ATP competes with cADPR for the binding site, resulting in the inhibition of the hydrolysis of cADPR.^86)^ [Enzyme-cADPR*] is suggested to be an enzyme-stabilized ADP-ribosyl oxocarbonium ion intermediate. The intermediate is thought to be attacked by H_2_O to form ADPR. Cys-119 and Cys-201 are essential for the hydrolysis reaction.^82)^ Glu-226 is essential for ADP-ribosyl cyclase.^289)^

The next issue concerns the mechanism by which the glucose stimulus induces the formation of cADPR. We^80,81)^ found that CD38, a 300-amino acid protein in human first recognized as a leukocyte antigen, was expressed in a variety of tissues including pancreatic β-cells. In 1993, CD38 was found to have both ADP-ribosyl cyclase, synthesizing cADPR from NAD^+^, and cADPR hydrolase to decompose cADPR to yield ADP-ribose.^80,107–111)^ We purified human CD38 protein and found that millimolar concentrations of ATP inhibited the cADPR hydrolase of CD38, competing with the substrate, cADPR.^80)^ Based on the competitive inhibition of the cADPR hydrolysis by ATP, cADPR and ATP appear to bind to the same site of CD38. By labeling the recombinant CD38 with an ATP analogue, 5′-*p*-fluorosulfonylbenzoyladenosine (FSBA), Tohgo *et al.*^86)^ identified the binding site for ATP and/or cADPR as the lysine-129 of CD38. Neither the Ala- nor Arg-129 mutant was labeled by FSBA nor catalyzed the hydrolysis of cADPR to ADP-ribose. Furthermore, the mutants did not bind cADPR, whereas they still used NAD^+^ as a substrate to form cADPR.^86)^ These results indicate that Lys-129 of CD38 participates in cADPR binding and ATP, produced in the process of glucose metabolism, competes with cADPR for the binding site, resulting in the inhibition of the cADPR hydrolase activity of CD38 and then in the accumulation of cADPR in β-cells (Fig. 9). Tohgo *et al.* also showed that cysteine-119 and -201 of CD38 were essential for the hydrolysis of cADPR to ADP-ribose.^82)^

### 5.3 Ca^2+^ release via ryanodine receptor (RyR) by cADPR.

cADPR is believed to activate a ryanodine receptor (RyR) to release Ca^2+^ from the intracellular stores, the endoplasmic reticulum.^104)^ We confirmed that type 2 RyR was expressed in rat pancreatic islets.^98,101)^ Experiments with rat islets revealed that cADPR did not bind directly to the RyR but acted on the receptor through a mediator such as FK506 (Tacrolimus)-binding protein 12.6 (FKBP12.6) to release Ca^2+^. The cellular target for FK506, one of the most widely used immunosuppressive agents, is thought to be FKBP12 and FKBP12.6. Rat FKBP12 is composed of 108 amino acids and is highly conserved among human, mouse, bovine, and rabbit FKBP12. Rat FKBP12.6 is also a 108-amino acid protein as are human and bovine FKBP12.6. Noguchi *et al.*^87)^ isolated microsomes from rat islets, carried out immunoblot analyses, and found that rat islet microsomes contained FKBP12.6, but did not contain FKBP12. Interestingly, cADPR was found to bind to FKBP12.6 at a *Kd* value of 35 nM. The cADPR-binding was inhibited by FK506, and neither structurally nor functionally related analogues of cADPR inhibited the cADPR binding to FKBP12.6. These results not only indicate that FKBP12.6 acts as a cADPR-binding protein, but also strongly suggest that cADPR is the physiological ligand of FKBP12.6 since FK506 does not normally exist in mammalian cells. As mentioned above, FKBP12.6 occurs in rat islet microsomes. When rat islet microsomes were treated with cADPR, FKBP12.6 was dissociated from the microsomes and moved to the supernatant, releasing Ca^2+^ from the intracellular stores. The microsomes that had been treated with FK506 or cADPR and were then devoid of FKBP12.6, did not show Ca^2+^ release by cADPR. These results and those from other experiments suggest that, when cADPR binds to FKBP12.6 in the RyR and causes the dissociation of FKBP12.6 from the RyR to form the FKBP12.6-cADPR complex, the RyR channel activity is thereby increased to release Ca^2+^ from the endoplasmic reticulum. When FK506 is present, cADPR cannot act on the RyR to release Ca^2+^, and the glucose-induced insulin secreting machinery ceases to function. Using FKBP12.6-deficient mice, Noguchi *et al.*^87,97)^ confirmed that FKBP12.6 plays a role in glucose-induced insulin secretion downstream of ATP production, independently of ATP-sensitive K^+^ channels, in pancreatic β-cells. In human, when FK506 was used as an immuno-suppressant in kidney transplantation, hyperglycemia was observed in 20–35% of the recipients.^112)^ The diabetogenic side effect of FK506 may be explained by the mechanism shown in Fig. 6. It should be also noted that, in the presence of calmodulin (CaM), islet microsomes become sensitized to cADPR at much lower concentrations for Ca^2+^ release, and the Ca^2+^ release is greatly increased.^83)^ Inhibitors for CaM-dependent protein kinase II (CaM kinase II) completely abolished the glucose-induced insulin secretion as well as the cADPR-mediated and CaM-activated Ca^2+^ mobilization. Western blot analysis revealed that rat microsomes contained CaM kinase IIα but did not contain CaM. When the active 30 kDa chymotryptic fragment of CaM kinase II was added to the microsomes, fully activated cADPR-mediated Ca^2+^ release was observed in the absence of CaM.^83)^ These results suggest that CaM kinase II is required to phosphorylate and activate the RyR for the cADPR-mediated Ca^2+^ release. As also shown in Fig. 6, cyclic AMP-dependent protein kinase (A-kinase) activated the cADPR-mediated Ca^2+^ release from islet microsomes.^94)^ In the absence of A-kinase, only a small amount of Ca^2+^ was released from the microsomes by low concentrations of cADPR. On the other hand, when the catalytic subunit of A-kinase was added to the islet microsomes, the Ca^2+^ release was sensitized at lower concentrations of cADPR. As incretin peptide hormones such as glucagon-like peptide-1 (GLP-1) and gastric inhibitory polypeptide (GIP, also known as glucose-dependent insulinotropic polypeptide) increase the intracellular concentrations of cyclic AMP to activate A-kinase and exchange protein activated by cAMP2 (EPAC2)/cAMP-guanine nucleotide exchange factor II,^113)^ it is quite possible the cADPR-mediated Ca^2+^ mobilization for insulin secretion is activated by CaM kinase II and A-kinase/EPAC2. Possibly, the activated kinases phosphorylate the RyR to sensitize the Ca^2+^ channel for the cADPR signal. Kim *et al.* also showed the involvement of cADPR in the enhancement of insulin secretion by GLP-1.^114)^

### 5.4 Deficiency of CD38 resulted in decreased intracellular cADPR, Ca^2+^ and insulin secretion.

To verify the role of the CD38-cADPR signal system in the regulation of insulin secretion, Kato *et al.*^84,91)^ created CD38 knockout (KO) and transgenic mice.

**Figure 10.**
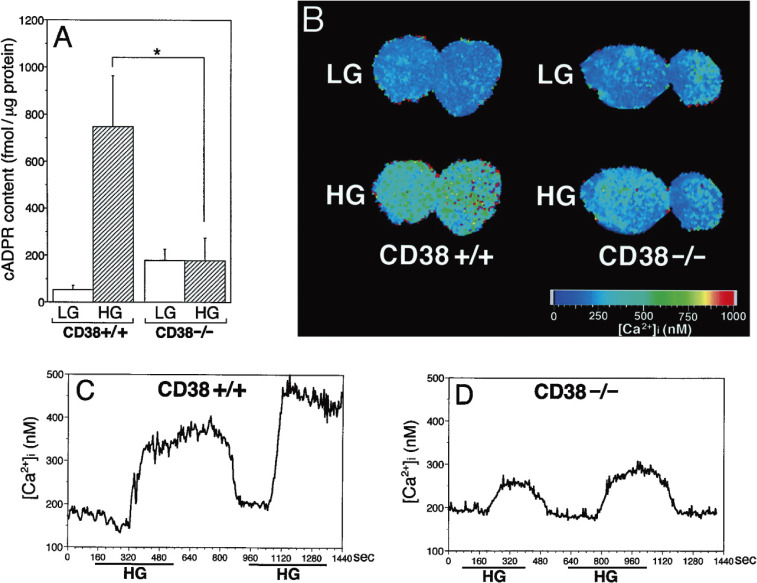
Measurement of cADPR content and glucose-induced [Ca^2+^]_i_ changes in CD38^+/+^ and CD38^−/−^ islets (adapted from Ref. 91). **A.** cADPR content in isolated islets under 2.8 mM glucose (LG) or 20 mM glucose (HG) concentrations, expressed as fmol per µg of protein of islet homogenate. n = 3–4 for each point. *, *P* < 0.05. **B.** Digital imaging of [Ca^2+^]_i_ in the islets. **C.** Changes in [Ca^2+^]_i_ in the CD38^+/+^ islets. A representative record from four experiments. **D.** Changes in [Ca^2+^]_i_ in the CD38^−/−^ islets. A representative record from seven experiments.

The glucose-induced increase in the cADPR content was greatly reduced, the Ca^2+^ concentration was impaired more than 50% in the KO mouse islets (Fig. 10), and the glucose-induced insulin secretion was decreased more than 50%.^91)^ CD38 KO mice showed impaired glucose tolerance, and the serum insulin level was lower than in the control. Further, whether the observed phenotype could be rescued by the pancreatic β-cell specific expression of CD38 cDNA was tested by crossbreeding transgenic mice carrying a human CD38 cDNA under the rat insulin promoter and the CD38 KO mice. By intercrossing their offspring, CD38 KO mice carrying the human CD38 transgene were generated. In the results, the human CD38 transgene ameliorated the glucose intolerance and the decreased insulin secretion. CD38 KO mice did not show insulin resistance, suggesting that the observed phenotype is indeed caused by the absence of CD38 in pancreatic β-cells.^91)^ The KO islets, however, responded normally to the extracellular Ca^2+^ influx stimulants tolbutamide and KCl to secrete insulin.^91)^ Thus, as shown in Fig. 6, the CD38-cADPR signal system as well as the ATP-sensitive K^+^ channel system appeared to function almost equally in insulin secretion by glucose stimulation.

This paradigm of insulin secretion based on the CD38-cADPR signal system has been supported by a wide body of evidence obtained in rats and mice. Recent results indicate that the CD38-cADPR signal system also functions in insulin secretion in human. As a result of a missense mutation (Arg140Trp) found in the CD38 gene in Japanese diabetic patients (4 in 31 Type 2 diabetic patients but none in 90 control subjects), the CD38 protein showed altered catalytic activities and a decreased production of cADPR.^115)^ The decreased function of the CD38 mutant may have contributed to the impairment of glucose-stimulated insulin secretion in Type 2 diabetes patients. It is also significant that circulating anti-CD38 autoantibodies were detected in 10–14% of Japanese^116)^ as well as Caucasian diabetic patients.^117–120)^ The autoantibody altered the *in vitro* enzymic activity of islet CD38, glucose-induced increase of cADPR levels, and insulin secretion.^116,117)^ These findings provide further support for the concept that the CD38-cADPR signal system functions in insulin secretion in human.

### 5.5 cADPR versus IP_3_ in intracellular Ca^2+^ release for insulin secretion (Fig. 11).

Since 1984, Berridge and Irvine^121)^ had proposed that IP_3_ induces Ca^2+^ release from the intracellular pool, the endoplasmic reticulum. They studied the Ca^2+^ release from the intracellular Ca^2+^ store in pancreatic acinar cells by IP_3_.^122)^

**Figure 11.**
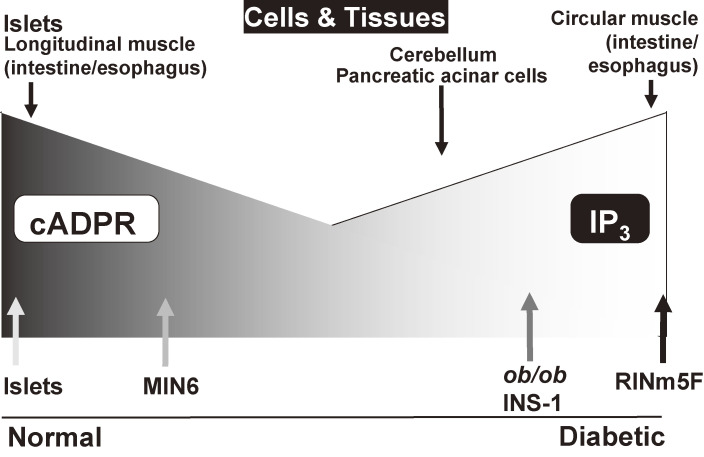
Alternative use of cADPR and IP_3_ as Ca^2+^ mobilizing second messengers in different types of cells/tissues and in normal and diabetic β-cells (adapted from Ref. 96). cADPR induces Ca^2+^ release from pancreatic islet microsomes but IP_3_ does not.^79)^ In cerebellum microsomes, both cADPR and IP_3_ induced Ca^2+^ release.^79)^ In rabbit small intestinal longitudinal muscle, but not circular smooth muscle, cADPR-dependent pathway participated in Ca^2+^ mobilization and muscle contraction.^290)^ Recently, in opossum esophagus, cADPR was found to induce the contraction of longitudinal smooth muscle, but not circular smooth muscle.^291)^

Concerning the Ca^2+^ release from the intracellular pool in pancreatic β-cells of the islets of Langerhans, the CD38-cADPR signal system proposed by the Okamoto group was the focus of intense debate.^88,104,107,123–126)^ Discrepant results were reported in diabetic β-cells such as *ob*/*ob* mouse islets and RINm5F cells, which have been often used for studying Ca^2+^ release. However, the Ca^2+^ release responses of these diabetic β-cell microsomes differed greatly from those of normal islet microsomes.^89)^ Microsomes from normal C57BL mouse islets released Ca^2+^ in response to cADPR, but scarcely in response to IP_3_. This response to cADPR was completely attenuated by the prior addition of 8-amino-cADPR. In *ob*/*ob* mouse islet microsomes, however, only a small amount of Ca^2+^ was released by cADPR, but much Ca^2+^ was released by IP_3_ (Fig. 8). All the RyR2 mRNAs in C57/BL islets were the islet-type^98,101)^ (see also Section 5.6). RINm5F cell microsomes also responded well to IP_3_ to release Ca^2+^ but did not respond to cADPR (Fig. 8). However, RINm5F cells are rat insulinoma-derived immortal cells that show almost no ability for glucose-induced insulin secretion. Concerning intracellular Ca^2+^ release channels, the mRNA expression of type 2 RyR, which is postulated to be a Ca^2+^ release channel for cADPR, was clearly detected in normal islets but not in *ob*/*ob* islets (Fig. 8A). In contrast, IP_3_ receptor (IP_3_R-1, IP_3_R-2, IP_3_R-4 and IP_3_R-5) mRNAs were not detected in normal islets but were clearly detected in *ob*/*ob* islets (Fig. 8B) and, although IP_3_R-3 mRNA was slightly detected in normal islets, the mRNA was significantly increased in *ob*/*ob* islets, consistent with the observation that IP_3_-induced Ca^2+^ mobilization preferentially worked in *ob*/*ob* islet microsomes. More importantly, the CD38 mRNA level was significantly decreased in *ob*/*ob* islets,^80)^ and CD38 mRNA was not expressed in RINm5F cells.^89)^ The decrease of CD38 mRNA in *ob*/*ob* islets may explain the low response in the cADPR content of β-cells by glucose stimulation. Decreased CD38 mRNA was also reported in islets of Goto-Kakizaki (GK) diabetic rats,^127)^ which showed impaired glucose-induced insulin secretion. These results show that the CD38-cADPR signal system for insulin secretion acts under normal physiological conditions but the IP_3_ system becomes dominant in diabetic β-cells such as *ob*/*ob* mouse islets and RINm5F cells (Fig. 11). In fact, it was confirmed by Rutter and co-workers and Ohta *et al.* using aequorin chimera that MIN6 cells, which retained the ability of glucose-induced insulin section and showed dramatic Ca^2+^ mobilization in response to cADPR^128–130)^ via the RyR despite the fact that no response to IP_3_ was observed. Kim and co-workers demonstrated that BALB/c mouse islets exhibited distinct increases in intracellular cADPR, Ca^2+^, and insulin secretion by glucose stimulation.^106)^

### 5.6 Alternative splicing of type 2 ryanodine receptor gene is essential for insulin production in islet β-cells.

**Figure 12.**
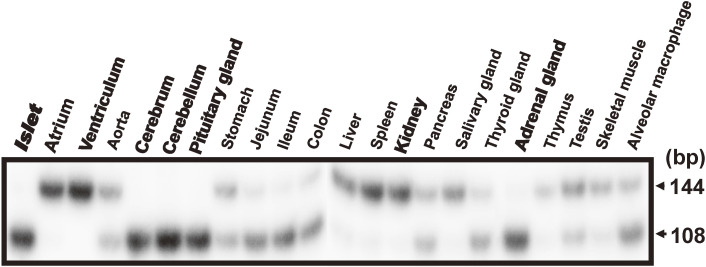
Splicing patterns of exon 75 in rat *RyR2* gene (adapted from Refs. 98 and 101). The 144 bp band contained exon 75 (cardiac-type RyR2) but the 108 bp band (islet-type RyR2) did not.

The CD38-cADPR signal system can be divided into two parts, the cADPR synthesis and metabolism and the Ca^2+^ release by cADPR from RyR (Fig. 6). There are three types of RyR in cells, RyR1 (type 1, skeletal type), RyR2 (type 2, cardiac type), and RyR3 (type 3, brain type). The three isoforms of RyR (RyR1, RyR2, and RyR3) are encoded by different genes and located in different chromosomal regions. RyR1 and RyR2 are predominantly expressed in skeletal muscles and cardiac muscles, respectively. On the other hand, RyR3 is ubiquitously expressed including in brain. Despite extensive physiological research, the type of RyR that is the target for cADPR has remained elusive. In pancreatic islets, RyR2 appeared to be expressed.^89,101,104)^ Takasawa, Kuroki *et al.*^98)^ isolated a novel RyR (islet-type RyR) from rat islet cDNA library, which was generated from *type 2 RyR* gene by alternative splicing of exons 4 and 75. The deduced protein of the cDNA was 4,947 amino acids with a calculated molecular weight of 562,291 daltons. These two regions, corresponding to the 7 amino acids in exon 4, and the 12 amino acids in exon 75 of cardiac RyR2, were not found in the rat islet *RyR* mRNA. Interestingly, neither exon 4 nor exon 75 was found in human and mouse islet *RyR2* mRNA. Further analysis of the alternative splicing patterns of *RyR2* mRNA in several rat tissues revealed that the islet-type *RyR* was expressed not only in islets but also in cerebrum, cerebellum, pituitary gland, and adrenal gland (Fig. 12). The cardiac-type *RyR* was expressed in atrium, ventriculum, and kidney. In many other tissues, both types are expressed. In the human *RYR2* gene, the tissue-specific alternative splicing pattern was essentially similar to that of the rat gene, and the islet-type *RYR* mRNA was also expressed in the brain as well as in islets.^98,101)^ Interestingly, the splice donor consensus “gt” was substituted by “gg” in the 0.8 kbp intron, whereas all the other splice donor and acceptor sequences conformed to the consensus “gt/ag rule” for splicing.^98,101)^

**Figure 13.**
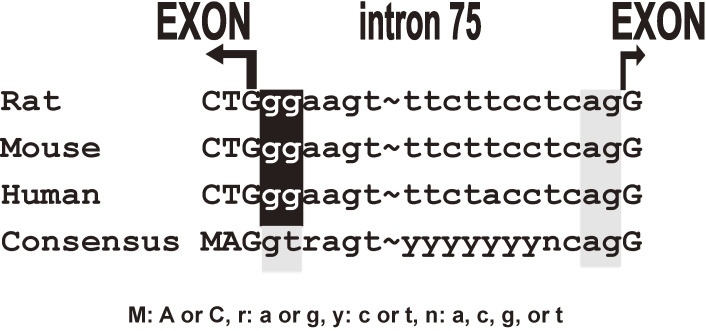
Usage of “gg” in splice donor sites of intron 75 in rat, mouse, and human *RyR2* (adapted from Ref. 101). Islet-type *RyR2* mRNA of rat, mouse, and human was generated by using “gg” as the splice donor site of intron 75.

**Figure 14.**
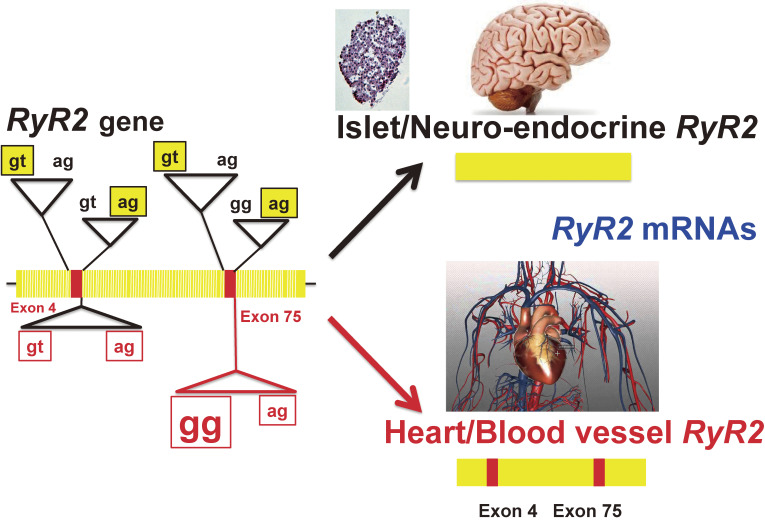
Tissue-specific alternative splicing of *RyR2* mRNA (adapted from Ref. 101). In pancreatic islet and neuro-endocrine cells, *RyR2* mRNA is generated/maturated using “gt/ag” splicing. On the other hand, *RyR2* mRNA is generated/maturated using not only the canonical “gt/ag” intron splicing site but also using the novel “**gg**/ag” site in heart and blood vessels. The exon 4 was also alternatively spliced by the “gt/ag” rule.^98,101)^ Therefore, the heart/blood vessel type *RyR2* mRNA can contain exon 4 and exon 75.

By RT-PCR analyses of human and mouse RNAs, the expression of (alternatively spliced) islet-type *RyR2* mRNA was also found in human and mouse islet RNAs. Furthermore, human and mouse islet-type *RyR* mRNA expressed in islets were also generated from the *RyR2* gene by alternative splicing. The splice donor consensus “gt” was also substituted by “gg”. As shown in Fig. 13, intron 75 of *RyR2* gene was spliced using “gg” as a donor for splicing instead of canonical “gt” in generating rat, mouse, and human *RyR2* mRNAs. *RyR2* gene for the CD38-cADPR Ca^2+^ signal system produced two different messenger RNAs by alternative splicing; one is for pancreatic islet β-cells and neuro-endocrine cells using “gt/ag” splicing, and the other is for heart and blood vessels using not only the canonical “gt/ag” intron splicing site but also using the novel “**gg**/ag” site. Therefore, the heart/blood vessel type RyR2 can contain exon 4 and exon 75 (Fig. 14).

The islet-type RyR caused a greater increase in the Ca^2+^ release by caffeine when expressed in HEK293 cells pretreated with cADPR, suggesting that the novel islet-type RyR was an intracellular target for the CD38-cADPR signal system in mammalian cells, playing many important physiological roles in the functioning of the cADPR-sensitive Ca^2+^ release.^101)^ Most recently, the islets-type *RyR2* was reported to be essential for insulin biosynthesis in human 1.1B4 insulin-producing cells via Ca^2+^ homeostasis.^131)^ The use of gg as a splice donor has not been reported in disease-free conditions.^132,133)^ Utilization of the gg splice donor was only reported in cholesteryl ester transfer protein gene in a patient with cholesteryl ester protein deficiency.^134)^

### 5.7 The CD38-cADPR signal system in other tissues and cells.

Although IP_3_ has been thought to be a second messenger for Ca^2+^ mobilization from intracellular stores, cADPR induces Ca^2+^ release from pancreatic islet microsomes but IP_3_ does not, as described above. In microsomes of the cerebellum, Ca^2+^ release is induced by both cADPR and IP_3_. It is, therefore, apparent that cells can utilize two second messengers, IP_3_ and cADPR, for Ca^2+^ mobilization, depending on the types of cells as well as differences in cellular conditions, physiological or pathological, performing a variety of cellular functions (Fig. 11). Recently, various physiological phenomena in animal, plant, and bacterial cells have been found to utilize this novel signal system.^93,94,99,101,135–150)^ In pancreatic acinar cells of CD38 KO mice, the acetylcholine-induced Ca^2+^ oscillation was greatly reduced or completely disappeared under a physiological concentration of acetylcholine.^144)^ Furthermore, acetylcholine induced cADPR formation in normal acinar cells but not in CD38 KO acinar cells. The IP_3_ formation was very small in the presence of a physiological concentration of acetylcholine and showed no difference between normal and CD38 KO cells. It appears likely that acetylcholine induces the cADPR formation via the G-protein coupled CD38 system.^145)^ In pancreatic β-cells, glucose is metabolized and induces the CD38-cADPR signal system to secrete insulin. In other cells, hormones and neurotransmitters may regulate the CD38-cADPR signal system in a receptor-coupled manner, such as in a G-protein-coupled manner, for various other types of physiological responses.^94,96)^

The possible involvement of the CD38-cADPR signal system in cardiovascular abnormalities has been reported. Myocardial hypertrophy was observed in male mice with null mutations of CD38, and FKBP12.6, a cADPR binding protein^146,147)^ and the altered stoichiometry of FKBP12.6 versus type 2 RyR as a cause of abnormal Ca^2+^ leak through RyR in heart failure in man.^148)^ As diabetic complications in the cardiovascular system are frequently observed, screening of the abnormalities in the CD38-cADPR signal system may provide a clue for the underlying molecular mechanism.

Jin *et al.* revealed that adult CD38 KO female and male mice showed marked defects in maternal nurturing and social behavior, respectively, with high locomotor activity.^149)^ Consistently, the plasma level of oxytocin was strongly decreased in CD38 KO mice. Oxytocin injection or lentiviral-vector-mediated delivery of CD38 in the hypothalamus rescued social memory and maternal care in CD38 KO mice but the introduction of CD38 Arg140Trp (rs1800561), which had been found in Japanese diabetic patients,^115)^ failed to recover the social memory and maternal care. Depolarization-induced oxytocin secretion and Ca^2+^ elevation in oxytocinergic neurohypophysial axon terminals were disrupted in the CD38 KO mice. These results indicate that CD38 has a key role in neuropeptide release, thereby critically regulating maternal and social behaviors, and may be an element in neurodevelopmental disorders. The mutation that caused tryptophan to replace arginine at amino acid residue 140 (CD38R140W; rs1800561[4693C>T])^115)^ was found in 0.6–4.6% of the Japanese population and was associated with autism spectrum disorder in a smaller case-control study.^151)^ In addition, maternal CD38R140W (rs1800561[4693C>T]) polymorphism was reported to be associated with an increased risk of admission to the neonatal intensive care unit due to preterm birth from Nara Medical University.^152)^ These reports strongly suggest that the CD38R140W (rs1800561[4693C>T]) polymorphism is a disease prone genotype, although the polymorphism is restricted in Mongoloid.^153)^

## 6 The Reg (Regenerating gene protein)-Reg receptor system for pancreatic β-cell regeneration of the islets of Langerhans

Insulin is synthesized in pancreatic β-cells in the islets of Langerhans and is the only hypoglycemic factor in human and animals. When food is eaten, large amounts of insulin are immediately secreted from β-cells, and then additional insulin is synthesized and stored in β-cells.^2,17,23)^ Therefore, the maintenance of the β-cell mass, as well as the insulin-synthesizing and -releasing ability of β-cells, as described in Sections 3, 4 and 5, is important in the process of insulin production.

According to the Okamoto model, streptozotocin- and alloxan-diabetes can be prevented by PARP inhibitors. In 1984, Yonemura *et al.*^154)^ successfully made 90% depancreatized rats and then we extended the model by proposing an integral function of poly ADP-ribosylation in cell regeneration.^36,96,154–167)^

### 6.1 Pancreatic β-cell regeneration in 90% depancreatized rats by the administration of PARP inhibitors.

**Figure 15.**
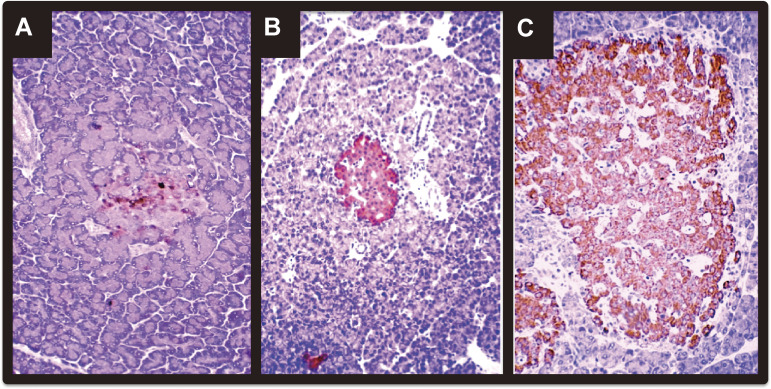
Proliferation of insulin-positive islet cells in the remaining pancreas of 90% depancreatized rats receiving daily injection of PARP inhibitor, nicotinamide (adapted from Refs. 164 and 292). **A.** Pancreatic tissue from 90% depancreatized rat receiving daily injection of saline. **B.** Pancreatic tissue from untreated control rat. **C.** Pancreatic tissue from 90% depancreatized rat receiving daily injection of nicotinamide. PARP inhibitor up-regulates *Reg*. Reg protein acts as an autocrine/paracrine growth factor for β-cell regeneration.^156,157,164,292)^

In 1984, we demonstrated that PARP inhibitors induced the regeneration of pancreatic β-cells in 90% depancreatized rats, thereby ameliorating the surgical diabetes.^154,156)^ Male Wistar rats were 90% depancreatized and, beginning 7 days before the partial pancreatectomy and continuing post-operatively, nicotinamide (0.5 g/kg body weight) or 3-aminobenzamide (0.05 g/kg body weight) was injected intraperitoneally every day. The 90% depancreatized rats exhibited glucosuria 1 to 3 months after the operation, but in the rats receiving nicotinamide or 3-aminobenzamide, the urinary glucose excretion level decreased markedly. Plasma glucose levels in rats receiving PARP inhibitors were also significantly lower than those in control 90% depancreatized rats.^36,154,160,163)^ The islets in the remaining pancreases of rats that had received the PARP inhibitors for 3 months were extremely large.^36,154,164)^ When the remaining pancreases were cytochemically stained, almost all the area of the enlarged islets was densely stained for insulin (Fig. 15), and an increase in the number of β-cells was found to be responsible for the increase in islet size (Table 1).^36,154,164)^ A small number of islet cells, however, were stained for insulin in the remaining pancreas of the control 90% depancreatized rats. On the other hand, cells stained for glucagon (α-cells) were localized only on the periphery of enlarged islets and their number was almost the same as that in islets in the parabiliary segment of normal rat pancreatic tissues. The number of cells stained for somatostatin (δ-cells) was also unchanged. These cytochemical findings indicate that it is the β-cell population that increased in the islets of the remaining pancreas of nicotinamide- and 3-aminobenzamide-treated rats, and thus the increased β-cell mass ameliorated the surgical diabetes.

### 6.2 Identification of *Reg* (*Regenerating gene*) in regenerating islets.

We isolated the rat regenerating islets and constructed a cDNA library. By differential screening of the regenerating islet-derived cDNA library, we found a novel gene expressed in regenerating islets. The cDNA had one large open reading frame encoding a 165-amino acid protein and the deduced protein has a signal sequence. We named the novel gene *Reg*, that is, ***re***generating ***g***ene, with the implication that the gene may be involved in islet regeneration.^157)^ We then isolated human *REG* gene.^157,168)^ Watanabe *et al.*^169)^ administered a recombinant rat Reg protein to 90% depancreatized rats and observed increased [^3^H]thymidine incorporation and frequent mitosis in islets of the remaining pancreas. On the 30th and 60th postoperative day, the fasting plasma glucose level of the rats that had received daily intraperitoneal injection of Reg protein (1 mg/kg/day) was significantly lower than that of the 90% depancreatized control rats. After 2 months, almost all the islets in the 90% depancreatized control rats were destroyed, whereas the islets of the remaining pancreas in the Reg protein-treated rats were enlarged and almost entirely stained for insulin.^169)^ Human REG protein administration also ameliorated diabetes in NOD mice with an increase in the β-cell mass.^170)^ These results indicate that Reg protein stimulates the regeneration and/or growth of pancreatic β-cells, thereby ameliorating animal diabetes.

Transgenic mice expressing *Reg* in β-cells showed increased [^3^H]thymidine incorporation in the islets.^171)^ The development of diabetes was significantly retarded in the *Reg* transgene-carrying NOD mice. On the other hand, *Reg* KO mice created by homologous recombination showed the decreased [^3^H]thymidine incorporation in the islets.^171)^ Further, when hyperplastic islets were induced by the injection of goldthioglucose, the islet sizes of *Reg* KO mice were significantly smaller than those from control *Reg*^+/+^ mice.^171)^

### 6.3 Reg protein signal.

As Reg protein was thought to act on pancreatic β-cells as an autocrine and/or paracrine growth factor, we tried to isolate a cDNA for the Reg protein receptor from a rat islet expression cDNA library using ^125^I-labeled rat Reg protein as a probe.^165)^ The isolated cDNA encoded a cell surface 919-amino acid protein, and a homology search revealed that the cDNA was a homologue to multiple exostoses-like gene, the function of which had hitherto been unknown. The COS-7 cells into which the cDNA had been introduced bound Reg protein with high affinity. When the cDNA was introduced into RINm5F β-cells, the transformants exhibited significant increases in the incorporation of 5′-bromo-2′-deoxyuridine as well as in the cell numbers in response to Reg protein, suggesting that the receptor mediated a growth signal of Reg protein for β-cell regeneration.^165)^

To determine what intracellular signal transduction events are induced in pancreatic β-cells by Reg protein, Takasawa *et al.* tested several signal transduction pathways in pancreatic β-cells by Reg protein stimulation using a PathDetect *trans*-reporting systems and found that activating transcription factor 2 (ATF-2) was activated by Reg protein stimulation.^167)^ The ATF-2 activation was also observed by the cotransfection of the Reg receptor expression plasmid instead of by Reg protein addition to the medium. Phosphorylation of ATF-2 at Thr-71 was increased by the stimulation of Reg protein. These results indicate that the Reg-Reg receptor system activates ATF-2, suggesting that genes under the control of ATF-2 play an important role in the cell cycle progression in pancreatic β-cells.

Cyclin D1 promoter activation by Reg protein was induced in a dose-dependent manner. In the electrophoretic mobility assay using cyclin D1 promoter sequence, Reg protein-stimulated β-cell nuclear proteins formed a specific complex with the ATF-2 binding site. Addition of the antibody against ATF-2 resulted in the formation of a supershift, suggesting the presence of ATF-2 in the complex.^167)^ The addition of phosphoinositide 3-kinase (PI(3)K) inhibitors LY294002 and wortmannin attenuated the Reg protein-induced ATF-2 phosphorylation/activation. Moreover, the immunoprecipitated PI(3)K phosphorylated ATF-2 in a time-dependent and dose-dependent fashion, suggesting that PI(3)K directly phosphorylated ATF-2 to activate the cyclin D1 promoter.^167)^

The *Reg* and *Reg*-related genes were isolated and revealed to constitute a multigene family, the *Reg* gene family^96,164)^ as later mentioned (Section 6.5). We made a knockout (KO) mice disrupting the gene of the *Reg* family, *Reg I*. The BrdU incorporation in *Reg I* KO islets without the addition of Reg I protein in the culture medium was reduced. The levels of phospho-ATF-2, cyclin D1 protein, and phospho-Rb in *Reg I*-deficient mouse islets were compared with those in normal littermates. Wild-type islets secreted Reg I in the culture medium and the phospho-Rb level in the islets was much higher than that in *Reg I* KO islets. This well fitted the decreased BrdU incorporation in *Reg I* KO islets. As expected, both the levels of phospho-ATF-2 and cyclin D1 were decreased in *Reg I* KO islets, whereas the levels of other proteins such as PARP^96,166)^ and CD38^80,91,96,101)^ as controls were unchanged.

**Figure 16.**
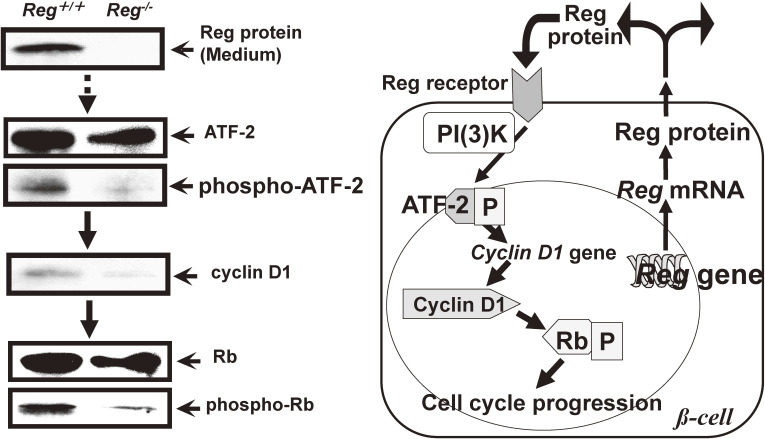
Decreases in phospho-ATF-2, cyclin D1, and phospho-Rb in *Reg*^*−/−*^ islets (adapted from Ref. 167). Islets were cultured without the addition of Reg I. Total protein from islets isolated from *Reg*^*+/+*^ or *Reg*^*−/−*^ mice was analyzed by Western blot. Reg expression in culture medium was also analyzed.

These results indicate that the activation of the cyclin D1 gene promoter in response to Reg I protein stimulation is mediated by the PI(3)K/ATF-2 signal transduction pathway in pancreatic β-cells, as shown in Fig. 16. Reg protein activated the cyclin D1 promoter for the cell cycle progression, and ATF-2 is an essential transcription factor in the process of Reg protein-induced cyclin D1 promoter activation. The PI(3)K activation is involved in the ATF-2 activation for cyclin D1 expression. It has been reported that disruption of the *CDK4* gene resulted in the development of insulin-deficient diabetes due to a reduction in the number of pancreatic β cells, and that the expression of a mutant CDK4, which escaped from the inhibitory regulation of CDK4, caused islets to become hyperplastic.^172)^ The results of the CDK4 disruption well explained the result that the islets from the *Reg I* KO mice showed reduced BrdU incorporation,^167)^ and that the islet hyperplasia induced by the goldthioglucose treatment was attenuated by *Reg I* gene disruption^171)^ because Reg protein induced a regulatory subunit of CDK4, cyclin D1, for the cell cycle progression (Fig. 16).

### 6.4 PARP inhibitors activate *Reg* gene transcription — A further development of the Okamoto model.

**Figure 17.**
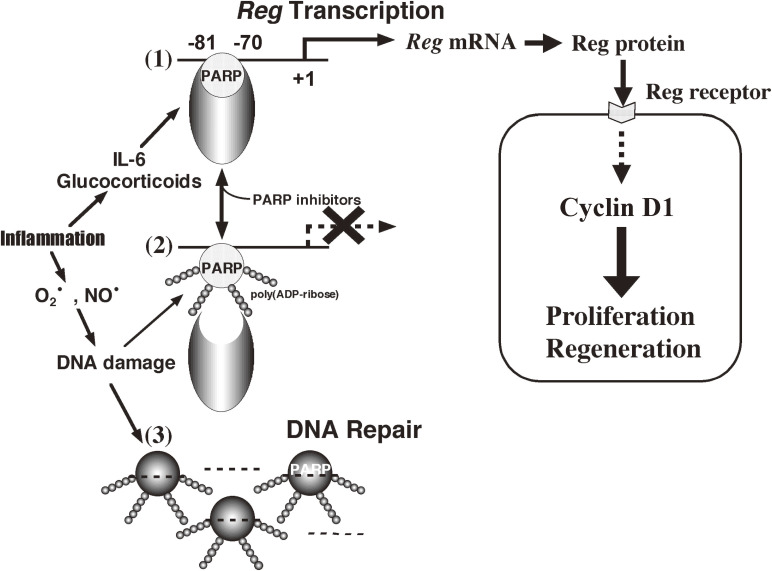
Roles of PARP in the *Reg* gene transcription and DNA repair (adapted from Ref. 166; see also Ref. 96). β-cells are affected by many agents such as immunological abnormalities, virus infections, irradiation, and chemical substances (see also Fig. 5), leading to local inflammation in and/or around pancreatic islets. (1) Inflammatory mediators such as IL-6 and glucocorticoids are produced in the inflammation process. IL-6/glucocorticoid stimulation induces the formation of an active transcriptional complex for *Reg*, in which PARP is involved, and when PARP is not auto-poly(ADP-ribosyl)ated in the presence of PARP inhibitors, the transcriptional complex is stabilized and the *Reg* gene transcription is maintained. (2) DNA damaging substances such as superoxide (O_2_^•^) and nitric oxide (NO^•^) are frequently produced in inflammatory processes. When the DNA is damaged, PARP senses DNA nicks and auto-poly(ADP-ribosyl)ates itself for the DNA repair. Once PARP is self-poly(ADP-ribosyl)ated, the formation of the *Reg* gene transcriptional complex is inhibited, interfering with the interaction between PARP and other nuclear proteins necessary for the active complex, and, therefore, the transcription of *Reg* gene stops. (3) When DNA is massively damaged, PARP is rapidly activated to repair the DNA^2,36)^ and the complex for *Reg* gene transcription is not formed at all.

Reg was induced in insulin producing pancreatic β-cells by inflammatory stimulation such as by interleukin-6 (IL-6)/glucocorticoids and IL-22^166,173–175)^ and acted as an autocrine/paracrine growth factor for β-cell regeneration via a cell surface Reg receptor (see Figs. 16 and 17)^165,167)^ to ameliorate experimental diabetes.^154,156,169)^ Further the *Reg* gene is only expressed during islet regeneration,^157,161)^ suggesting that the regeneration and proliferation of pancreatic β-cells for the increase of the cell mass were primarily regulated by the *Reg* gene expression. In this context, Akiyama *et al.*^166)^ found that *Reg* gene was activated by IL-6, dexamethasone, and PARP inhibitors. The combined addition of IL-6 and dexamethasone increased the *Reg* mRNA level, and further addition of nicotinamide or 3-aminobenzamide increased the mRNA even more. Progressive deletion of the 5′-flanking region of rat *Reg* gene revealed that the region between nucleotides -81 and -70 “TGCCCCTCCCAT” is essential for the *Reg* gene promoter activity. In gel mobility shift assays with the *Reg* promoter, the intensity of the band, which was detected in the nuclear extracts of RINm5F cells treated with IL-6, dexamethasone and/or nicotinamide, was correlated with the luciferase activity, and the protein binding to the sequence was revealed to be PARP.^166)^ The inhibition of PARP activity was shown to facilitate *Reg* transcription by preventing the excessive self-poly(ADP-ribosyl)ation of PARP and the consequent dissociation of PARP from the active transcriptional DNA/protein complex. The transcriptional regulation by PARP activity and the transcription-regulating PARP-binding sequences were subsequently described in other genes such as chemokine (C-X-C motif) ligand 1,^176)^ nuclear factor κ-light-chain-enhancer of activated B cells-regulated genes,^177)^ α-synuclein,^178)^ V-type proton ATPase 116 kDa subunit a isoform 3,^179)^ B-cell lymphoma 6 protein,^180)^ tatrate-resistant acid phosphatase,^181)^ vimentin,^182)^ cyclooxygenase-2,^183)^ hemochromatosis protein,^184)^ cellular communication network factor 2,^185)^ micro opioid receptor,^186)^ microsomal epoxide hydrolase,^187)^ FoxO1^188)^ and PARP.^189)^ So far, the novel view that PARP acts as a transcriptional regulator has been established.

Thus, as shown in Fig. 17, inflammatory mediators, IL-6 and glucocorticoids, induce the formation of an active transcriptional complex involving PARP for *Reg* gene, and the *Reg* gene transcription therefore proceeds. However, during inflammation, superoxide (O_2_^•^) and nitric oxide (NO^•^) are produced, causing DNA damage. In this situation, PARP is activated by DNA nicks to repair the DNA. Then, PARP poly(ADP-ribosyl)ates PARP itself, the poly(ADP-ribose) chains on the PARP protein inhibit the formation of the active transcriptional complex, and the *Reg* gene transcription is halted. In the presence of PARP inhibitors such as nicotinamide, the PARP is not poly(ADP-ribosyl)ated, the transcriptional complex is stabilized, and the *Reg* gene transcription proceeds. Therefore, PARP inhibitors maintain the function of PARP as a transcription factor for β-cell regeneration. This can account for the islet regeneration found in 90% depancreatized rats treated with PARP inhibitors^154)^ and also supports the notion that the restriction of β-cell replication is relieved by PARP inhibitors.^36)^ When DNA is massively damaged, PARP is rapidly activated to repair the DNA, as described in Section 4, and the complex for *Reg* gene transcription is not formed at all.

### 6.5 *Reg* gene family and its expression in a variety of cells for regeneration/proliferation: Death from early cancer is predicted by the presence of transcripts of the *REG* gene family.

**Figure 18.**
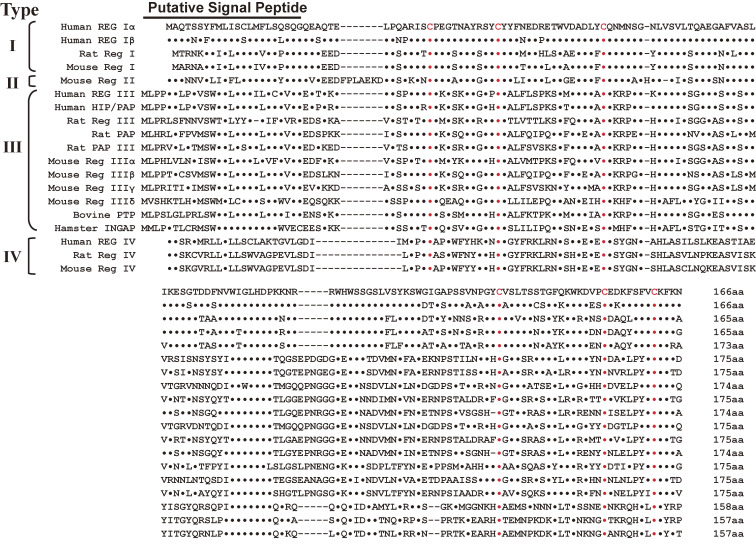
Alignment of amino acid sequences of the *Reg* gene family. Based on the primary structures of the encoded proteins (adapted from Refs. 96, 195 and 293), the members of the *Reg* gene family are grouped into four subclasses, types I, II, III and IV. *Dots* indicate amino acids identical to human REG Iα. *Dashes* indicate gaps for maximal alignment. Six conserved cysteines in the mature proteins are indicated by *red*. Type I, II, and III *Reg* genes are clustered in a restricted region of the same chromosome.^195,196,198,293)^ Human *REG*-related sequence (*RS*) is omitted from the alignment because human *RS* is a pseudogene.^168)^ Recently, hamster *Reg III*β (XM_021231969.2), *Reg III*γ (NM_001281579.1) and *Reg IV* (XM_021235470.2), shrew *Reg III*α (XM_006169192.2), *Reg III*γ (XM_006169191.2) and *Reg IV* (XM_006166291.1), rabbit *Reg III*γ (XM_002709697.2) and *Reg IV* (XM_002715670.3), canine *Reg III*α (NM_001002945.3) and *Reg IV* (XM_038626000.1), guinea pig *Reg III*β (XM_003468964.1) and *Reg III*γ (XM_003468904.4), bovine *Reg IV* (NM_001076986.1), porcine *Reg III*γ (XM_005662419.3) and *Reg IV* (NM_001190251.1), equine *Reg III*γ (XM_001498171.6), ferine *Reg IV* (XM_023259045.1), kangaroo *Reg III*β (XM_013031828.1), suncus *Reg III*α (LC606672), bat *Reg III*α (XM_033125205.1), *Reg III*γ (XM_024558600.1) and *Reg IV* (XM_024570607.1), chicken *Reg IV* (NM_001277527.1), quail *Reg IV* (XM_015869386.1), and frog *Reg I*β (XM_002934582.4) and *Reg IV* (XM_004915988.3) were isolated. The 6 cysteines are completely conserved among the spices in the primary structure.

The *Reg* and *Reg*-related genes were isolated and revealed to constitute a multigene family, the *Reg* gene family.^96,164,190,191)^ Based on the primary structures of the Reg proteins, the members of the family are grouped into four subclasses, types I, II, III, and IV (Fig. 18). In human, four *REG* family genes, *i.e.*, *REG I*α,^157,168)^ *REG I*β,^192)^ *REG*-related sequence (*RS*),^168)^ *HIP*^193)^/*PAP*,^194)^ and *REG III*^195)^ are tandemly ordered in the 95 kbp region of chromosome 2p12,^196)^ whereas *REG IV* is located on chromosome 1.^197)^ In the mouse genome, all the *Reg* family genes except for *Reg IV*, *i.e.*, *Reg I*, *Reg II*, *Reg III*α, *Reg III*β, *Reg III*γ, and *Reg III*δ were mapped to a contiguous 75 kbp region of chromosome 6C,^198)^ whereas *Reg IV* was mapped on chromosome 3. Type I and mouse Type II Reg proteins are expressed in regenerating islets^157,190)^ and are involved in β-cell regeneration.^169,199–208)^ Reg family proteins have been suggested to be involved in cellular proliferation in exocrine pancreatic cells,^209,210)^ gastrointestinal cells,^211–222)^ *Helicobacter pylori*-infected gastric mucosa,^223,224)^ hepatic cells,^225–229)^ cardiovascular cells,^230–232)^ salivary ductal cells,^233–235)^ bone and muscle cells,^236,237)^ and neuronal cells.^238)^ Importantly, mouse Reg III was shown to be a Schwann cell mitogen that accompanied the regeneration of motor neurons,^239)^ and Reg protein functions as a neurotrophic factor for motor neurons.^240,241)^ Reg was also shown to mediate gastric mucosal proliferation in rats.^242,243)^

**Figure 19.**
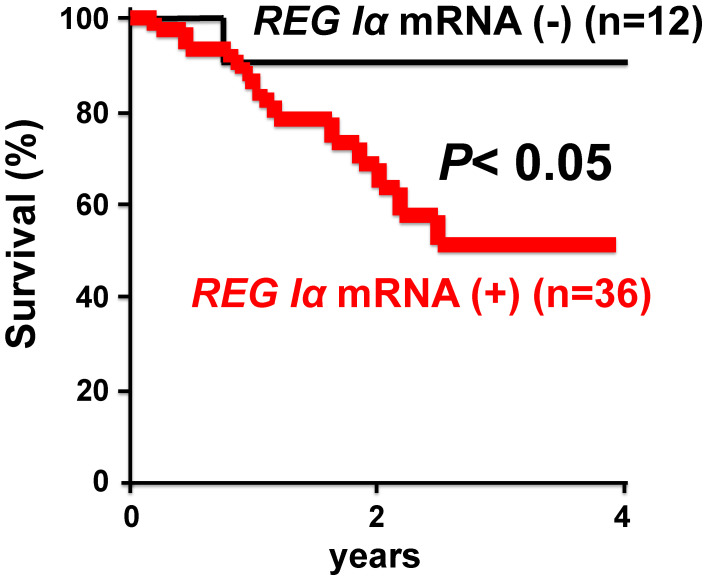
(Color online) Death from early cancer is predicted by the presence of transcripts of the *REG* gene family: Disease-free survival of patients with differentiated gastric adenocarcinomas (adapted from Ref. 245). The *red* (*thick*) *line* represents patients with tumors that expressed *REG I*α mRNA (n = 36), whereas the *black* (*thin*) *line* represents patients with tumors that did not express *REG I*α mRNA (n = 12).

In 2000, a group of St. James’s University Hospital reported that death from early colorectal cancer was predicted by the presence of transcripts of the *REG* gene family.^244)^ In 2003, Yonemura *et al.*^245)^ showed that the expression of the *REG I*α gene is closely related to the infiltrating property of gastric carcinomas, and may be a prognostic indicator of differentiated adenocarcinoma of the stomach (Fig. 19). Since then, a correlation between *REG* family gene expression and cancer prognosis has been reported.^246–256)^ These observations suggest that the *Reg* gene family is involved in a variety of cell types including cancer cells for regeneration/proliferation.

## 7 Concluding remarks

Based on the results of Sections 2, 3, and 4 we described that PARP activation, which was induced by damaged DNA, caused the NAD^+^ depletion to form poly(ADP-ribose) and the decreased β-cell function such as proinsulin synthesis, resulting in necrotic cell death (Fig. 5). Since induction of insulin biosynthesis in islet β-cells is achieved at the level of translation (as discussed in Section 3), an immediate decrease in insulin biosynthetic activity cannot be attributed to DNA damage itself. Rather, the β-cell, it may reasonably be assumed, commits suicide in its attempt to repair the DNA strand breaks.^1,2,35–37)^

In Section 5, we described that cADPR formation from NAD^+^ was essential for cell functions, such as insulin secretion in pancreatic β-cells. An increase in the intracellular Ca^2+^ concentration, which then triggers insulin secretion, has conventionally been explained by Ca^2+^ influx from extracellular sources. On the other hand, in 1993, we showed a novel mechanism of insulin secretion in which the Ca^2+^ release from the endoplasmic reticulum, an intracellular Ca^2+^ pool, induced insulin secretion (Fig. 6).^79,80)^ Rojas *et al.* examined the Ca^2+^ influx from extracellular sources and the intracellular pool in human β-cells, and showed that 42–75% of the increase in the intracellular Ca^2+^ concentration by glucose stimulation was due to the release of Ca^2+^ from the intracellular stores.^257)^ Thus, the importance of Ca^2+^ release from the endoplasmic reticulum in insulin secretion by glucose has been recognized.

**Figure 20.**
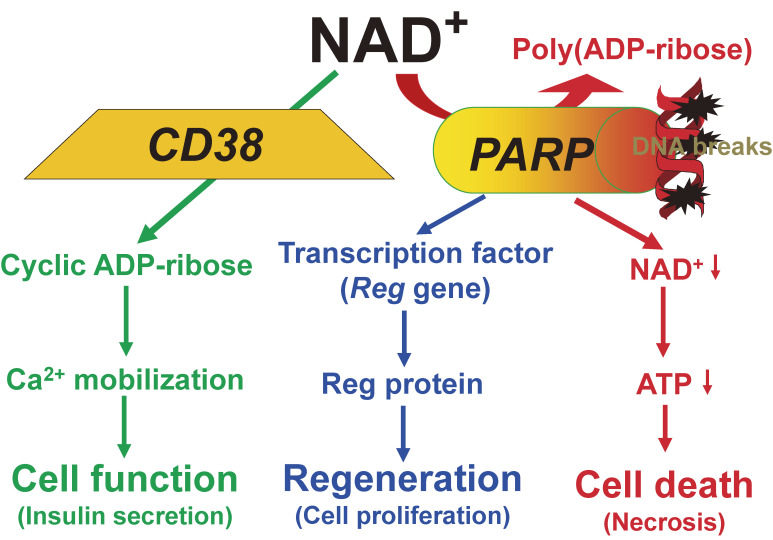
A possible model of cell destination via CD38 and PARP. NAD^+^ is catalyzed to form cADPR by CD38. Ca^2+^ mobilization is caused by cADPR, leading to cell functioning such as insulin secretion. DNA breaks induce PARP activation, leading to cell death (necrosis) via NAD^+^ depletion. When PARP acts as a transcription factor, *Reg* genes/proteins are expressed for cell regeneration/proliferation. It should be noted here that nicotinamide, a PARP inhibitor, prolongs survival of primary cultured hepatocytes without involving loss of hepatocyte-specific functions.^294)^

In Section 6, we described that PARP inhibitors induced the regeneration of pancreatic β-cells in 90% depancreatized rats, thereby ameliorating the surgical diabetes.^154,156)^ Then, we isolated ***Reg*** (***Re****generating* ***g****ene*) from the regenerating islet-derived cDNA library^157,160)^ and established the Reg-Reg receptor system for the cell regeneration/proliferation (Figs. 16 and 17). Further, PARP was found to act as a transcription factor for *Reg* gene, and the active transcription complex for *Reg* gene was not formed when PARP was activated and auto-poly(ADP-ribosyl)ated (Fig. 17). Therefore, the inhibition of the PARP activity by PARP inhibitors results in at least three important events in the cell (Fig. 20): PARP inhibitors prevent cell death (Fig. 5), maintain the formation of a second messenger, cADPR, to achieve the cell function, and keep PARP active as a transcription factor for cell regeneration or proliferation (Fig. 17).

**Figure 21.**
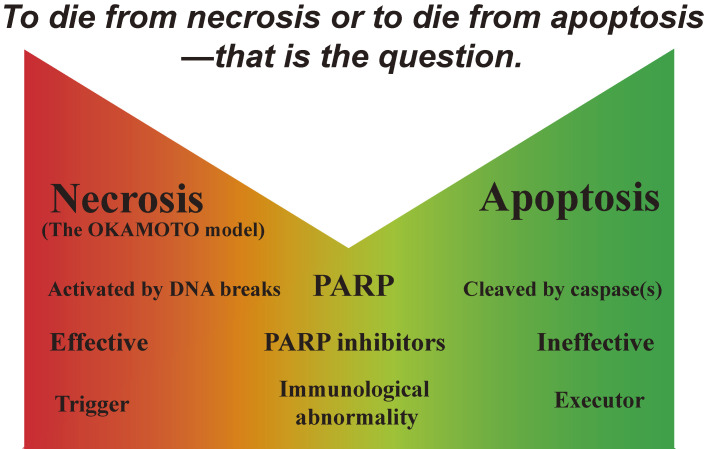
Comparison of two types of cell death, necrosis and apoptosis (adapted from Ref. 100). The cell death by PARP activation described in Section 4 is thought to occur through necrosis. In apoptotic cell death, PARP is cleaved by caspases and inactivated. Therefore, PARP inhibitors can be effective in preventing necrosis but ineffective in preventing apoptosis. “Whether to die from necrosis or to die from apoptosis” may depend on the severity and duration of the cell damage, differences in death signals, and the species of cells. A report from Bhardwaj’s laboratory suggests that dendritic cells distinguish between two types of cell death, with necrosis providing a control that is critical for the initiation of immunity.^283)^ Therefore, immunological abnormalities, which are frequently observed in Type 1 diabetes, may be triggered by the preceding necrotic cell death, and then cause apoptotic death of β-cells (see also Fig. 22).

**Figure 22.**
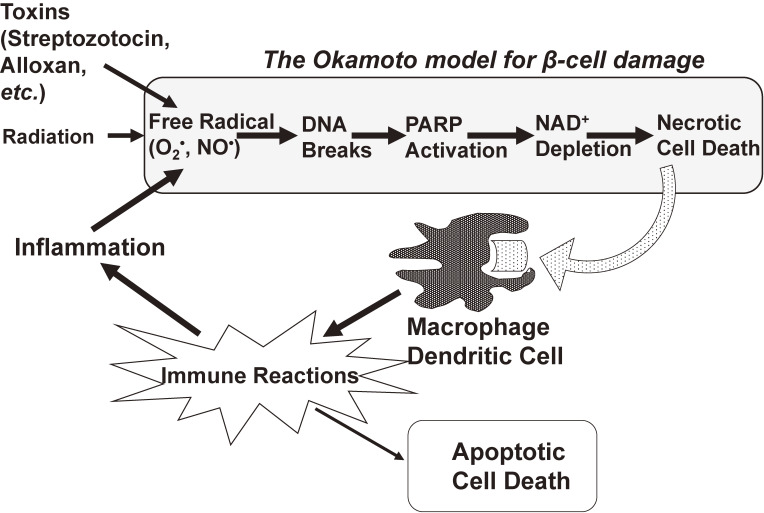
The Okamoto model for necrotic cell death (adapted from Ref. 96). The Okamoto model, originally proposed as a unifying model for β-cell damage and its prevention, well explains both how autoimmunity for β-cell necrosis is initiated and how necrotic cell death is involved in various diseases in many tissues other than β-cells. Under physiological conditions, apoptotic cell death constitutively occurs for renewal and maintenance in animal bodies, whereas necrotic cells initiate and enhance (auto)immune reactions under pathological conditions. On the other hand, apoptotic cells recognized by macrophages/dendritic cells inhibit phlogistic (auto)immune responses. For the initiation of massive and pathological apoptotic cell death, necrotic cell death, which triggers autoimmune responses in macrophages/dendritic cells, is required.

In Section 4, PARP inhibitors were shown to prevent necrotic β-cell death. In 1987, Vague *et al.*^258)^ reported that nicotinamide, a PARP inhibitor, could extend the remission phase of insulin-dependent Type 1 diabetes. Then, clinical trials with nicotinamide have been performed by several groups in the world. The results suggested that the nicotinamide treatment may be promising one for the prevention and the cure of Type 1 diabetes,^259–264)^ whereas in another study nicotinamide was reported to be somewhat ineffective to prevent Type 1 diabetes.^265)^ Concerning this, Uchigata *et al.*^7)^ have already reported that it is essentially important when the nicotinamide administration against Type 1 diabetes should be started: before, just after, or while β-cells are being damaged. It is also important that nicotinamide^266,267)^ can protect the necrotic cell death but cannot protect the apoptotic cell death as shown in Figs. 21 and 22. Actually, pretreatment of islet grafts with nicotinamide prevented their deterioration in a mouse islet transplantation model.^268)^ Furthermore, in human islet isolation for islet transplantation, nicotinamide significantly improved islet yields in both the University of Wisconsin and the two-layer method preservation protocols.^269)^ PARP inhibitors were also shown in Section 6 to induce *Reg* gene expression by stabilizing the transcriptional complex for *Reg* gene, resulting in the β-cell regeneration and neogenesis from the progenitor cells such as pancreatic duct cells.^270)^ In generating insulin-producing endocrine β-cells from mouse embryonic stem cells and human induced pluripotent stem cells, nicotinamide was found to be an indispensable factor for differentiation/proliferation into insulin-producing endocrine β-cells.^271,272)^ It is, therefore, possible that the amelioration of diabetes observed in the clinical nicotinamide trials could be caused not only by the prevention of necrotic β-cells but also by the induction of β-cell regeneration/neogenesis. Furthermore, as described in Section 5, because cADPR is produced from NAD^+^ by ADP-ribosyl cyclase of CD38, and because PARP is the major enzyme for NAD^+^ degradation/consumption, the inhibition of PARP by inhibitors such as nicotinamide may maintain the cADPR formation from NAD^+^ for β-cells to achieve their functions, such as glucose-stimulated insulin secretion.

Therefore, PARP inhibitors and their derivatives appear to be one of the promising therapeutic approaches for diabetes treatment, although some toxic effects such as reversible liver toxicity,^273)^ teratogenicity,^274)^ growth-retardation,^275)^ and oncogenicity^35,36,276)^ were reported in animal models at very high doses of the inhibitor. However, it has been revealed that there is no evidence in man of most of the toxic effects of nicotinamide that have been reported in animal models.^264)^

Some experiments using cytokines as β-cytotoxic agents showed that nicotinamide did not effectively inhibit human β-cell death.^277,278)^ However, there are two kinds of cell death, necrosis and apoptosis. PARP activation results in necrotic cell death^36,37,100,155,279)^ whereas, in apoptotic cell death, PARP is cleaved by caspases and inactivated.^280–282)^ Therefore, PARP inhibitors can prevent necrosis but not apoptosis (Fig. 21). The type of cell death, whether necrosis or apoptosis, would depend on the severity and duration of the insult, differences in signaling pathways, and the type of cell. It has been suggested that dendritic cells/macrophages can distinguish between the two types of cell death, and that necrosis may provide the critical control for the initiation of immunity^283,284)^ upon recognition of necrotic cells, and the proinflammatory responses of activated macrophages are increased. In contrast, apoptotic cells have a strong inhibitory effect on phlogistic macrophage responses. Thus, immunological abnormalities, which are frequently observed in Type 1 diabetes, may be triggered by preceding necrotic cell death, followed by apoptotic death of β-cells (Fig. 22).

More recently, roles of the NAD^+^ preservation in the islet β-cell survival and of the CD38-cADPR signal system in insulin secretion (described in Sections 4 and 5) with Imeglimin ((6*R*)-*N*^2^,*N*^2^,6-Trimethyl-3,6-dihydro-1,3,5-triazine-2,4-diamine monohydrochloride) have been recognized.^285)^ Imeglimin is the first in a new class of oral anti-Type 2 diabetes agent^286)^ and is scheduled to be launched in 2021 in Japan for the first time in the world. 

Never advance anything which cannot be proved in a simple and decisive fashion.

—Louis Pasteur (1888)—

## Figures and Tables

**Table 1.** Cell numbers in the parabiliary segment of rat pancreatic tissues

Treatment of rats	**β-cells**(number/cm^2^)	**α-cells**(number/cm^2^)	**δ-cells**(number/cm^2^)
Normal untreated	2086 ± 507	285 ± 85	191 ± 42
90% Depancreatized	442 ± 482	359 ± 120	185 ± 72
90% Depancreatized+nicotinamide(0.5 g/kg per day)	3204 ± 1412**	310 ± 207	133 ± 119
90% Depancreatized+3-aminobenzamide(0.05 g/kg per day)	2286 ± 1173*	310 ± 161	92 ± 42

*P < 0.05 *vs.* 90% depancreatized rats (control); **P < 0.01 *vs.* 90% depancreatized rats (control).

The number of cells was determined in the parabiliary segment of at least five rats and expressed as the mean ± SD. Adapted from Refs. 156 and 164.

## Non-standard abbreviation list

A-kinase: cyclic AMP-dependent protein kinase
alloxan: 2,4,5,6-tetraoxohexahydropyrimidine
ATF-2: activating transcription factor 2
cADPR: cyclic ADP-ribose
CaM: calmodulin
CaM kinase II: calmodulin-dependent protein kinase II
CDK: cyclin-dependent kinase
FK506: Tacrolimus (Tsukuba macrolide immunosuppressant)
FKBP: FK506-binding protein
FSBA: 5′-*p*-fluorosulfonylbenzoyladenosine
GLP-1: glucagon-like peptide-1
IL-6: interleukin-6
IP_3_: inositol 1,4,5-trisphosphate
KO: knockout
NO: nitric oxide
NOD: non-obese diabetic
NOS2: type 2 nitric oxide synthase
NOS2: type 2 nitric oxide synthase
PARP: poly(ADP-ribose) polymerase/synthetase
PI(3)K: phosphoinositide 3-kinase
Reg: Reg protein
*Reg*: *Regenerating gene*
RyR: ryanodine receptor
streptozotocin: 2-deoxy-2-(3-methyl-3-nitrosoureido)-D-glucopyranose
